# Evolution of the Araliaceae family involved rapid diversification of the Asian Palmate group and *Hydrocotyle* specific mutational pressure

**DOI:** 10.1038/s41598-023-49830-7

**Published:** 2023-12-15

**Authors:** Jong-Soo Kang, Vo Ngoc Linh Giang, Hyun-Seung Park, Young Sang Park, Woohyeon Cho, Van Binh Nguyen, Hyeonah Shim, Nomar Espinosa Waminal, Jee Young Park, Hyun Hee Kim, Tae-Jin Yang

**Affiliations:** 1 Department of Agriculture, Forestry and Bioresources, Plant Genomics and Breeding Institute, Research Institute of Agriculture and Life Science, College of Agriculture and Life Sciences, Seoul National University, Seoul, 08826 South Korea; 2 Faculty of Pharmacy, University of Medicine and Pharmacy at Ho Chi Minh City, Ho Chi Minh City, 700000 Vietnam; 3 Department of Integrative Biological Sciences and Industry, Sejong University, Seoul, South Korea; 4 Faculty of Biology, Dalat University, Dalat, 670000 Vietnam; 5 Department of Life Science, Chromosome Research Institute, Sahmyook University, Seoul, 01795 South Korea; 6 Leibniz Institute of Plant Genetics and Crop Plant Research (IPK), 06466 Seeland, Gatersleben, Germany

**Keywords:** Molecular evolution, Phylogenetics

## Abstract

The Araliaceae contain many valuable species in medicinal and industrial aspects. We performed intensive phylogenomics using the plastid genome (plastome) and 45S nuclear ribosomal DNA sequences. A total of 66 plastome sequences were used, 13 of which were newly assembled in this study, 12 from new sequences, and one from existing data. While Araliaceae plastomes showed conserved genome structure, phylogenetic reconstructions based on four different plastome datasets revealed phylogenetic discordance within the Asian Palmate group. The divergence time estimation revealed that splits in two Araliaceae subfamilies and the clades exhibiting phylogenetic discordances in the Asian Palmate group occurred at two climatic optima, suggesting that global warming events triggered species divergence, particularly the rapid diversification of the Asian Palmate group during the Middle Miocene. Nucleotide substitution analyses indicated that the Hydrocotyloideae plastomes have undergone accelerated AT-biased mutations (C-to-T transitions) compared with the Aralioideae plastomes, and the acceleration may occur in their mitochondrial and nuclear genomes as well. This implies that members of the genus *Hydrocotyle*, the only aquatic plants in the Araliaceae, have experienced a distinct evolutionary history from the other species. We also discussed the intercontinental disjunction in the genus *Panax* and proposed a hypothesis to complement the previously proposed hypothesis. Our results provide the evolutionary trajectory of Araliaceae and advance our current understanding of the evolution of Araliaceae species.

## Introduction

Araliaceae, the ginseng family, is a highly diversified family containing approximately 45 genera and 1500 species; several morphological characteristics of the Araliaceae, such as flower morphology, overlap with those of the closely related Apiaceae^1–5^. The Araliaceae were previously merged into the Apiaceae (Umbelliferae) owing to their similar morphology, but eventually previous studies treated the Araliaceae as an independent family^2,6–8^. This family consists of two subfamilies: the species-rich Aralioideae, and the Hydrocotyloideae, containing two genera (*Hydrocotyle* and *Trachymene*). Species in the family Araliaceae are widely distributed in tropical and subtropical regions of both the northern and southern hemispheres, with less diversity in temperate regions^1,9^. These species are well known as useful biological resources for both medicinal (*Panax*, *Aralia*, *Eleutherococcus*, *Heteropanax*, *Kalopanax*, *Tetrapanax*, and *Fatsia*) and ornamental (*Hedera*, *Dendropanax*, and *Schefflera*) purposes, with many studies having investigated the medicinal properties of Araliaceae species^10–16^.

Phylogenetic questions around the Araliaceae remain despite several previous studies investigating them^1,2,4,17–20^. Phylogenetic studies using the internal transcribed spacer (nrITS) region of the nuclear ribosomal DNA (nrDNA) and several plastid markers suggest four major groups within the Aralioideae subfamily—the Asian Palmate group, the *Aralia*–*Panax* group, the Greater *Raukaua* group, and the *Polycias*–*Pseudopanax* group—and clearly support the monophyly of the two subfamilies, Aralioideae and Hydrocotyloideae^1,2,4,17–20^. However, phylogenetic discordance has emerged between different phylogenetic trees and regarding the poly- or paraphyly of the genera *Schefflera*, *Pseudopanax*, *Aralia*, and *Polyscias*^1,2,4,17–20^. This highlights the limitations, such as polytomy, of phylogenetic analyses using a few DNA barcoding regions, indicating that genome-based phylogenetic studies are required to shed light on phylogenetic relationships within the Araliaceae.

Plastids, which have an endosymbiotic origin, are organelles specific to plant cells and contain their own genomes. Unlike the mitochondrial genomes (mitogenomes), which are another endosymbiotic organelle genome of plant cells and have complicated genome structures, plastid genomes (plastomes) have a conserved quadripartite structure and are around 150 kb in size^21,22^. In most land plants, plastomes contain two copies of an inverted repeat (IR) region separating a large single-copy (LSC) region and a small single-copy (SSC) region^21^. Reports of complete plastid genomes and phylogenomic studies using plastome data have rapidly increased with high-throughput sequencing technologies. Although maternally inherited plastid DNA often exhibited phylogenetic discordance with biparentally inherited nuclear DNA, plastome data are still widely used for revealing phylogenetic relationships and many studies have demonstrated the utility of plastome data for resolving relationships among various species^23–27^. In the Araliaceae, numerous plastomes have been used to develop molecular markers for species identification and to reveal phylogenetic relationships in the genus *Panax* and the Asian Palmate group; *Panax* species include popular medicinal plants such as Korean ginseng, and phylogenetic relationships within the Asian Palmate group remain unclear^28–31^. In addition, evolution of the *Panax* species has been examined because of their intercontinental disjunction pattern between East Asia and North America^29,32–35^. However, the plastome evolution and phylogenetic relationships of the Araliaceae have been poorly studied at the family level using plastome data. The nrDNA consists of two transcription units, 45S and 5S, thousands of copies of which are tandemly repeated in the nuclear genome^36,37^. The tandemly repeated nrDNA structure results in conserved sequence mutations, which contribute to the ease of sequencing and use for phylogenetic studies^38,39^.

Low-coverage whole-genome sequencing allows stimultaneous assembly of maternally inherited plastome and biparentally inherited nrDNAs^40,41^. In this study, we newly assembled the complete plastomes and 45S nrDNAs, including nrITS regions, of 13 species (12 Araliaceae species and one Apiaceae species) and compared these with previously reported Araliaceae plastomes. Furthermore, we inferred phylogenetic relationships among the Araliaceae using previously reported plastome and nrDNA sequences from NCBI GenBank and explored plastome evolution within the family Araliaceae.

## Results

### Plastid genome and 45S nrDNA assemblies

Twelve species were newly sequenced using Illumina sequencing platforms (Supplementary Table S1), and the genome sequence of *Hydrocotyle vulgaris* (ERX5309978) was downloaded from the NCBI SRA database. We successfully assembled 13 plastomes as circular genomes comprising two copies of the IR region (25,060–25,969 bp) separated by the LSC (84,198–86,630 bp) and the SSC (17,869–18,708 bp) regions (Supplementary Fig. S1A, Supplementary Table S1). The mean coverage of the assembled plastomes ranged from 227 × in *Hedera helix* to 2108 × in *Centella asiatica* (Apiaceae) with no gaps. All newly assembled plastomes were similar to previously reported Araliaceae and Apiaceae plastomes in genome structure and gene content, even in the IR and SC boundaries, with *Hydrocotyle vulgaris* having the smallest plastome (153,026 bp) and *Schefflera arboricola* the largest one (156,704 bp). The GC content of plastomes ranged from 37.7% in *Hydrocotyle vulgaris* and *Centella asiatica* to 38.1% in the two *Aralia* plastomes (Supplementary Table S1), similar to previously reported Araliaceae plastomes. All the plastomes assembled in this study contained 80 protein-coding genes, 31 tRNAs, and four rRNAs with 115 unique genes overall (Supplementary Table S2). Seventeen genes were duplicated in the IR region, giving a total of 132 genes in the Araliaceae plastomes. Eighteen genes contained one or two introns: 15 genes contained one intron and three genes (*rps12*, *clpP*, and *ycf3*) contained two introns. Six of the intron-containing genes were tRNAs (Supplementary Table S1).

The complete 45S nrDNA transcription unit assemblies, including 18S, 5.8S, 15S, and two internal transcribed spacers (ITS1 and ITS2), were almost identical in length, with mean sequence coverage ranging from 305 × in *Hydrocotyle vulgaris* to 3094 × in *Kalopanax septemlobus* (Supplementary Fig. S1B; Supplementary Table S1). The 5.8S rRNA sequence was 160 bp in all species, whereas the lengths of 18S, ITS1, ITS2, and 26S varied by species: 1807–1808 bp for 18S rRNA, 222–228 bp for ITS1, 231–237 bp for ITS2, and 3395–3399 bp for 26S rRNA. The GC content was more than 53%, ranging from 53.6% in *Hydrocotyle vulgaris* to 55.8% in *Centella asiatica* (Supplementary Table S1).

### Phylogenetic relationships in the Araliaceae

**Figure 1:**
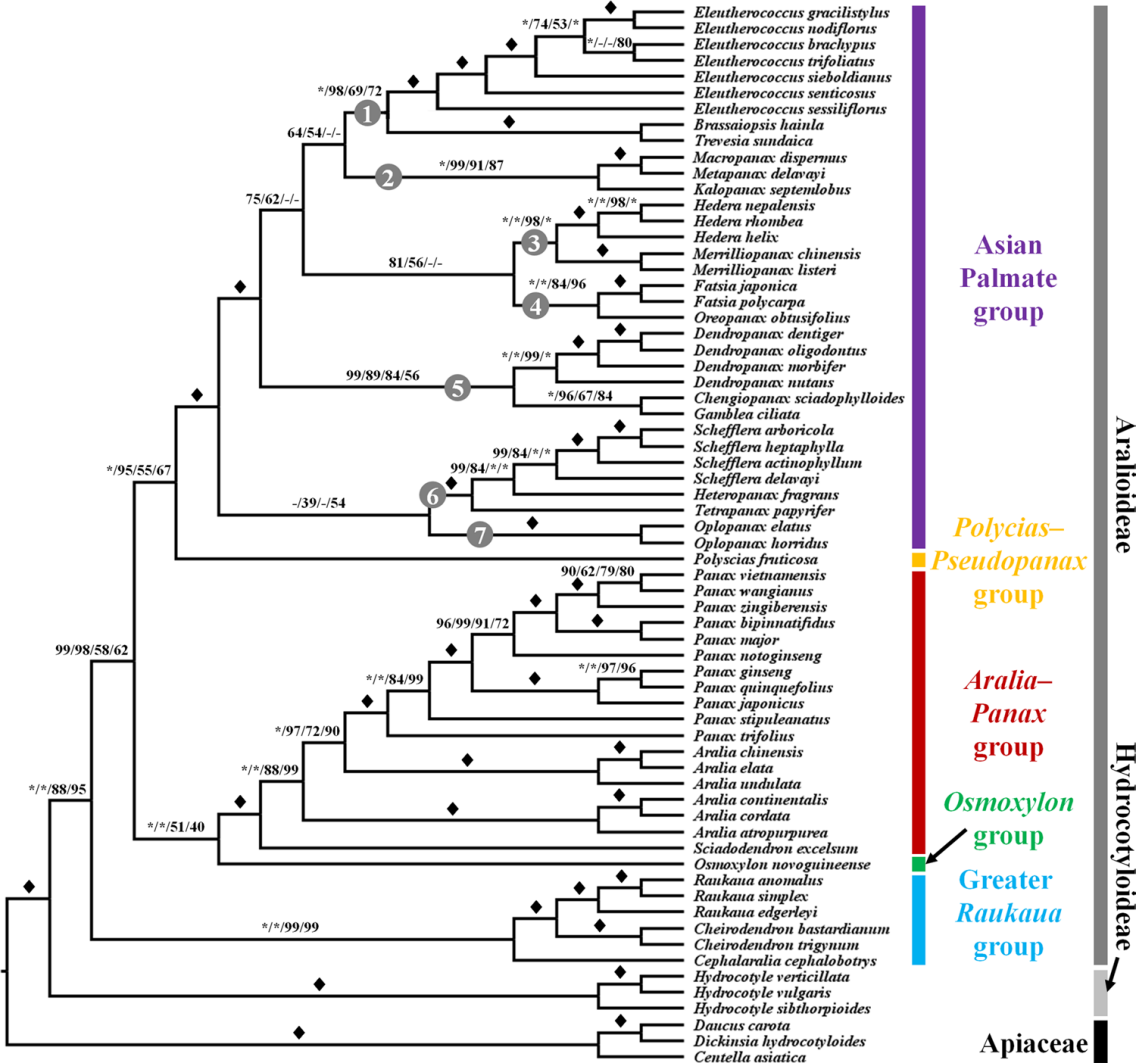
A summary of Araliaceae phylogenies inferred from four different datasets. Bootstrap values calculated by maximum-likelihood analyses of whole-plastome sequences, concatenated protein-coding gene sequences, first and second sites of CDSs, and amino acid sequences, respectively, are shown near branches. Solid diamonds indicate full support (100%) from four different datasets, and asterisks indicate full support from the corresponding dataset. Hyphens indicate missing values owing to different results for the topology of the corresponding dataset. Clades marked by numbers in gray circles correspond to the clades shown in Fig. 2.

**Figure 2:**
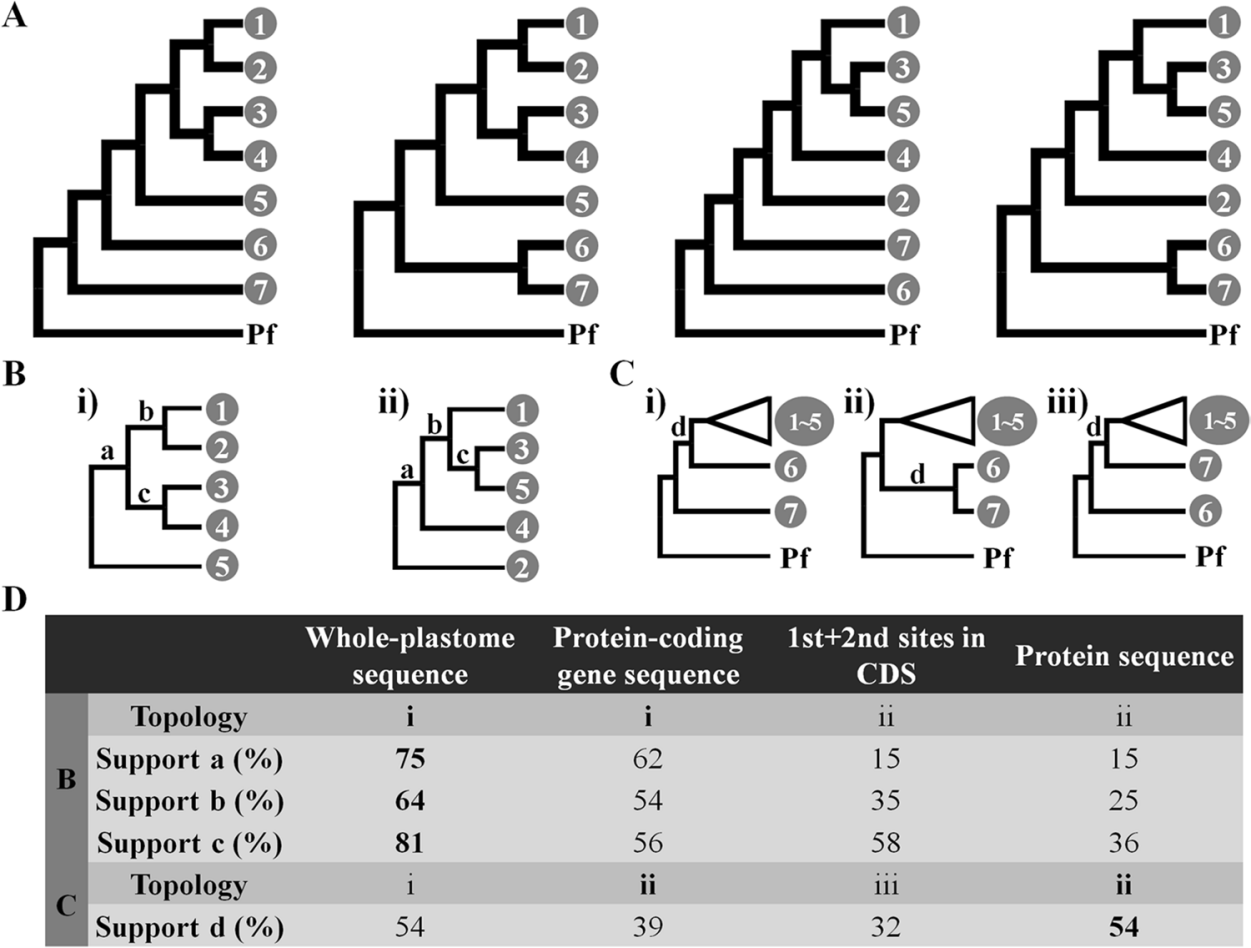
Conflicting phylogenetic results for the Asian Palmate group in the Araliaceae. (**A**) Four topologies inferred from four different datasets (whole-plastome sequence, protein-coding gene sequence, first and second sites in CDS, and protein sequence); simplified topologies for each conflict are illustrated in B, C, D, and E. Detailed information for clades marked by numbers in gray circles can be found in Fig. 1. (**B**) Conflicts within the Asian Palmate Core. (**C**) Conflict of the *Oplopanax* position. (**D**) Topologies supported and bootstrap values estimated by maximum-likelihood analyses based on four different datasets. Topologies correspond to those in (**B** and **C**). The greatest bootstrap value among the four phylogenies is indicated in bold. Pf: *Polyscias fruticosa*.

We performed phylogenetic reconstructions based on plastome data using four different data matrices: whole-plastome sequences, concatenation of 78 protein-coding gene sequences (CDSs), first and second sites of the 78 CDSs, and translated protein sequences (Fig. 1, Supplementary Figs.S2–S5). Phylogenetic results from the four data matrices indicated that the two subfamilies within the Araliaceae, Aralioideae and Hydrocotyloideae, are monophyletic, while monophyly of the three major groups in the Aralioideae, the Asian Palmate group, the *Aralia*–*Panax* group, and the Greater *Raukaua* group, was well resolved (Fig. 1, Supplementary Figs.S2–S5). However, the genus *Aralia* was not monophyletic in all four phylogenies (Fig. 1, Supplementary Figs.S2–S5), similar to previous studies^1,2,4,17–19^. We were not able to examine the monophyly of the *Polycias*–*Pseudopanax* group or the *Osmoxylon* group because we had only one sample from each group. Topologies within the Asian Palmate group differed using the four data matrices. Within the Asian Palmate group, we observed phylogenetic discordance in the positions of the *Macropanax*–*Metapanax*–*Kalopanax* clade (Clade 2), the *Fatsia*–*Oreopanax* clade (Clade 4), and the *Dendropanax*–*Chengiopanax*–*Gamblea* clade (Clade 5), and in the monophyly of the *Schefflera*–*Heteropanax*–*Tetrapanax* clade (Clade 6) and the *Oplopanax* clade (Clade 7) (Fig. 2A–C). The clades 2, 4, and 5 were each located in two positions; however, the topology inferred from whole-plastome sequences and protein-coding gene sequences had stronger branch support than the topology inferred from the first and second sites of the 78 CDSs or the translated protein sequences (Fig. 2B,D). Monophyly of clades 6 and 7 was inferred from the protein-coding gene sequences and the translated protein sequences, although the branch support values were relatively low (Fig. 2C,D).

**Figure 3:**
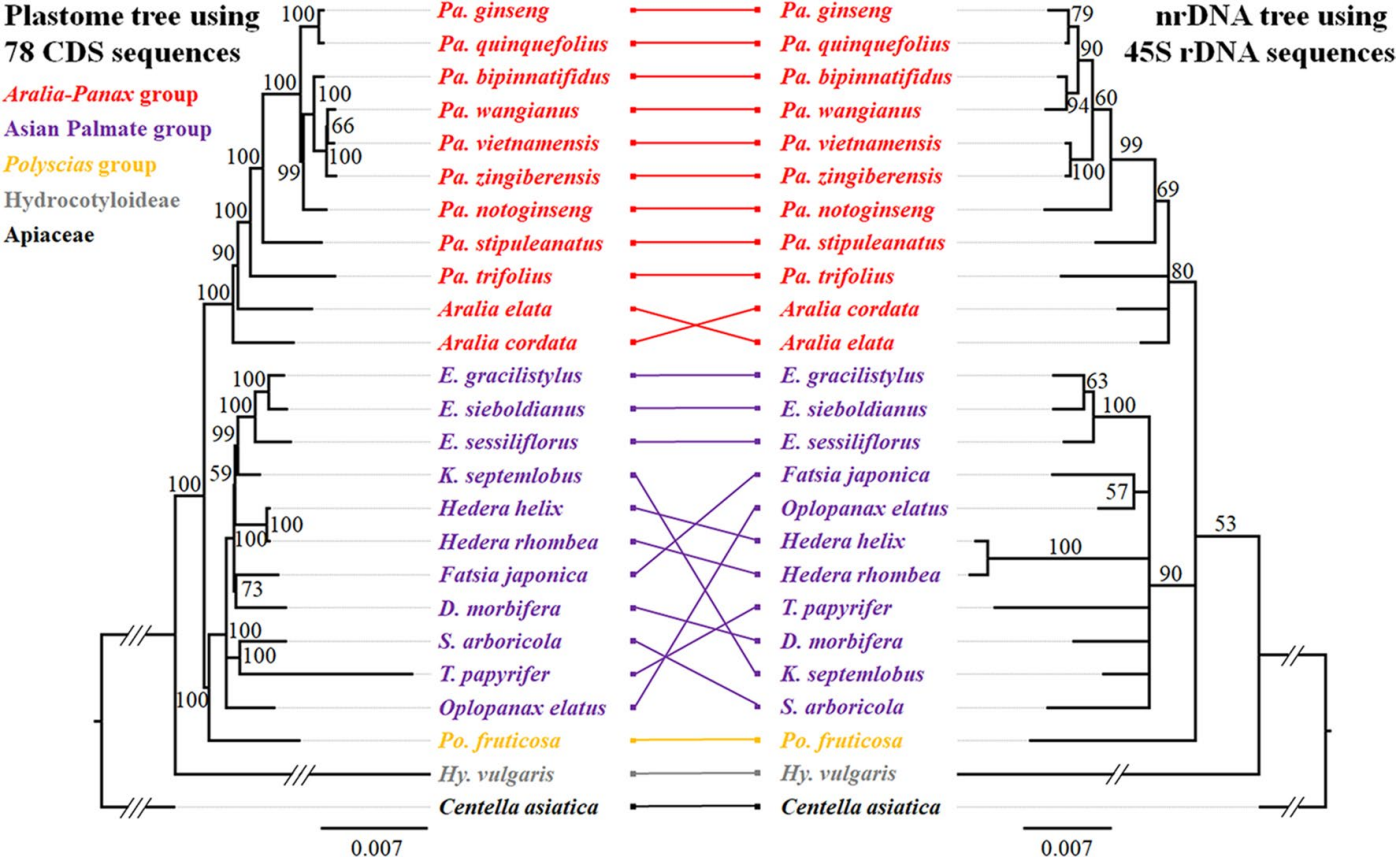
Comparison between plastome-based and nrDNA trees of the Araliaceae. nrDNA sequences of 25 species were available for phylogenetic reconstruction. Phylograms of plastome (left) and nrDNA (right) trees inferred by the maximum-likelihood method using RAxML; bootstrap support values greater than 50% are indicated above branches, and nodes with less than 50% support were collapsed. Each group is marked in a different color. Long branches are indicated with slashes and shortened; each slash represents one scale bar. *E*.: *Eleutherococcus*, *K*.: *Kalopanax*, *D*.: *Dendropanax*, *S*.: *Schefflera*, *T*.: *Tetrapanax*, *Po*.: *Polyscias*, *Pa*.: *Panax*, *Hy*.: *Hydrocotyle*.

The phylogeny based on 45S nrDNA sequences, including 18S, ITS-1, 5.8S, ITS2, and 26S regions, indicated strong support for monophyly of the Asian Palmate group and the *Aralia*–*Panax* group, with bootstrap support values of 90% and 80%, respectively (Fig. 3). However, phylogenetic relationships within the Asian Palmate group conflicted with those in the plastome-based phylogeny and remained unclear owing to a lack of bootstrap support (less than 50%), indicating polytomy (Fig. 3). The monophyly of the genus *Panax* also collapsed because of the position of *Panax trifolius*. *P. trifolius*, distributed in North America, is known to be the most basal group of the genus *Panax*, which was supported by our plastome-based phylogenies (Fig. 1, Supplementary Figs.S2–S5); however, phylogenetic positions of *P. trifolius*, *Aralia cordata* and *A. elata* were unclear with low support (less than 50%) in the nrDNA phylogeny (Fig. 3). While *P. stipuleanatus* occupied a consistent position as sister to all *Panax* species (except *P. trifolius*) in the two phylogenies, phylogenetic positions of other *Panax* species were incongruent (Fig. 3). *P. notoginseng* was sister to a clade of four *Panax* species, *P. bipinnatifidus*, *P. wangianus*, *P. vietnamensis*, and *P. zingiberensis*, in the plastome-based phylogeny; however, it was sister to a clade of these four *Panax* species and two tetraploid *Panax* species, *P. ginseng* and *P. quinquefolius*, in the nrDNA phylogeny (Fig. 3). *Polyscias fruticosa* also showed a difference in phylogenetic position between the plastome and nrDNA phylogenies, being sister to the Asian Palmate group in the plastome phylogeny but showing uncertain relationships with both the Asian Palmate and *Aralia*–*Panax* groups in the 45S nrDNA phylogeny (Fig. 3). Consequently, we inferred the species network of the family Araliaceae by considering the topologies and branch support values obtained from the plastome-based phylogenies using the four data matrices as well as the 45S nrDNA phylogeny, and this network was used to estimate divergence times of Araliaceae species by constraining the topology (Fig. 1).

### Nucleotide substitution analyses

Using the plastome sequence of *Centella asiatica* (Apiaceae) as a reference, we estimated nucleotide substitution rates using 78 plastid genes of 63 Araliaceae plastomes. Synonymous (*d*_*S*_) and nonsynonymous (*d*_*N*_) nucleotide substitution rates of three *Hydrocotyle* plastomes in the subfamily Hydrocotyloideae were relatively higher than those of plastomes in the subfamily Aralioideae (Table 1), and differences in nucleotide substitution rates were supported statistically by analyses of variance (ANOVA) and Bonferroni *post-hoc* tests (*P* < 0.001) (Supplementary Fig. S6). Compared with the Aralioideae plastomes, the *d*_*S*_ was relatively faster than the *d*_*N*_ in the Hydrocotyloideae plastomes (Table 1), leading to a lower *d*_*N*_/*d*_*S*_ ratio (Supplementary Fig. S6). Within the Aralioideae, the *d*_*S*_ of the *Aralia*–*Panax* group was statistically higher (*P* < 0.001) than those of the Asian Palmate group and the Greater *Raukaua* group. The difference in *d*_*S*_ between the *Aralia*–*Panax* group and *Osmoxylon*/*Polyscias* groups was not significant owing to having just single samples of *Osmoxylon* and *Polyscias* (Supplementary Fig. S6). *Hydrocotyle* plastomes in the Hydrocotyloideae showed a statistically significant difference from other groups in the *d*_*N*_ analysis, whereas differences among the five groups in the Aralioideae were not supported in any case (Supplementary Fig. S6). The *Aralia*–*Panax* group had an equivalent *d*_*N*_ and high *d*_*S*_ compared with the other four groups in the Aralioideae, resulting in a relatively low *d*_*N*_/*d*_*S*_ ratio (*P* < 0.001), and this phenomenon was also confirmed in the *Hydrocotyle* plastomes (*P* < 0.001).

**Table 1:** Transition and transversion between subfamilies and among four groups in the Araliaceae family.

	Nucleotide substitution rate		Number of transition		Number of transversion			
	*d*_*S*_	*d*_*N*_	T–C	C–T	T–G	G–T	A–T	G–C
Aralioideae	0.1083 ± 0.0563	0.0176 ± 0.0199	1023.9 ± 24.3	760.9 ± 24.5	234.3 ± 21.21	325.8 ± 11.88	172.1 ± 9.08	198.4 ± 6.97
Hydrocotyloideae (genus *Hydrocotyle*)	**0.1696 ± 0.0743**	**0.0259 ± 0.0290**	**1252.0 ± 15.6**	**1314.3 ± 40.4**	**435.3 ± 13.05**	**450.0 ± 28.69**	**275.0 ± 11.53**	**278.3 ± 5.69**
*Aralia*–*Panax* group	0.1115 ± 0.0586	0.0175 ± 0.0198	1045.4 ± 23.4	777.7 ± 13.2	258.1 ± 13.22	324.6 ± 12.16	170.9 ± 7.89	207.3 ± 4.41
Asian Palmate group	0.1067 ± 0.0550	0.0176 ± 0.0199	1013.8 ± 17.6	754.8 ± 23.8	222.2 ± 15.59	326.1 ± 12.44	172.2 ± 9.59	194.0 ± 3.30
Greater *Raukaua* group	0.1041 ± 0.0537	0.0176 ± 0.0205	1022.7 ± 23.5	736.3 ± 18.2	233.3 ± 6.44	329.2 ± 10.80	173.7 ± 11.62	196.2 ± 3.25

*Polyscias* and *Osmoxylon* were excluded from this analysis because the mean and standard deviation could not be calculated from single samples. *d*_*S*_: synonymous substitution rate; *d*_*N*_: nonsynonymous substitution rate.

*Each value represents the mean and standard deviation of the substitution (mean ± standard deviation), with the greatest value shown in bold.

Using the *Centella asiatica* plastome as the standard, we analyzed transitions and transversions to determine which nucleotide substitution types are increased in the Hydrocotyloideae plastomes showing significantly higher substitution rates compared with Aralioideae plastomes (Table 1; Supplementary Fig. S7). The most common substitutions in protein-coding regions of Araliaceae plastomes were transitions, with the most common transition type being a T-to-C (A-to-G) transition, occurring in a range from 987 sites in *Oreopanax obtusifolius* to 1270 sites in *Hydrocotyle sibthorpioides* (Table 1; Supplementary Fig. S7). All the plastomes of the subfamily Aralioideae had more T-to-C transitions than C-to-T (G-to-A) transitions; however, plastomes in the subfamily Hydrocotyloideae had more C-to-T transitions than T-to-C transitions. The three *Hydrocotyle* (Hydrocotyloideae) plastomes had relatively higher numbers of both transitions and transversions than the Aralioideae plastomes, but the number of C-to-T transitions in the Hydrocotyloideae plastomes was approximately double that in the Aralioideae plastomes (Table 1; Supplementary Fig. S7). This increase in C-to-T transitions was found in all protein-coding genes (not any specific gene) used in the analyses of substitution rate and type, and appeared to be a major driver of the accelerated nucleotide substitution rates of Hydrocotyloideae plastomes compared with Aralioideae plastomes (Table 1; Supplementary Figs. S6, S7).

### Divergence time and ancestral distribution estimation

**Figure 4:**
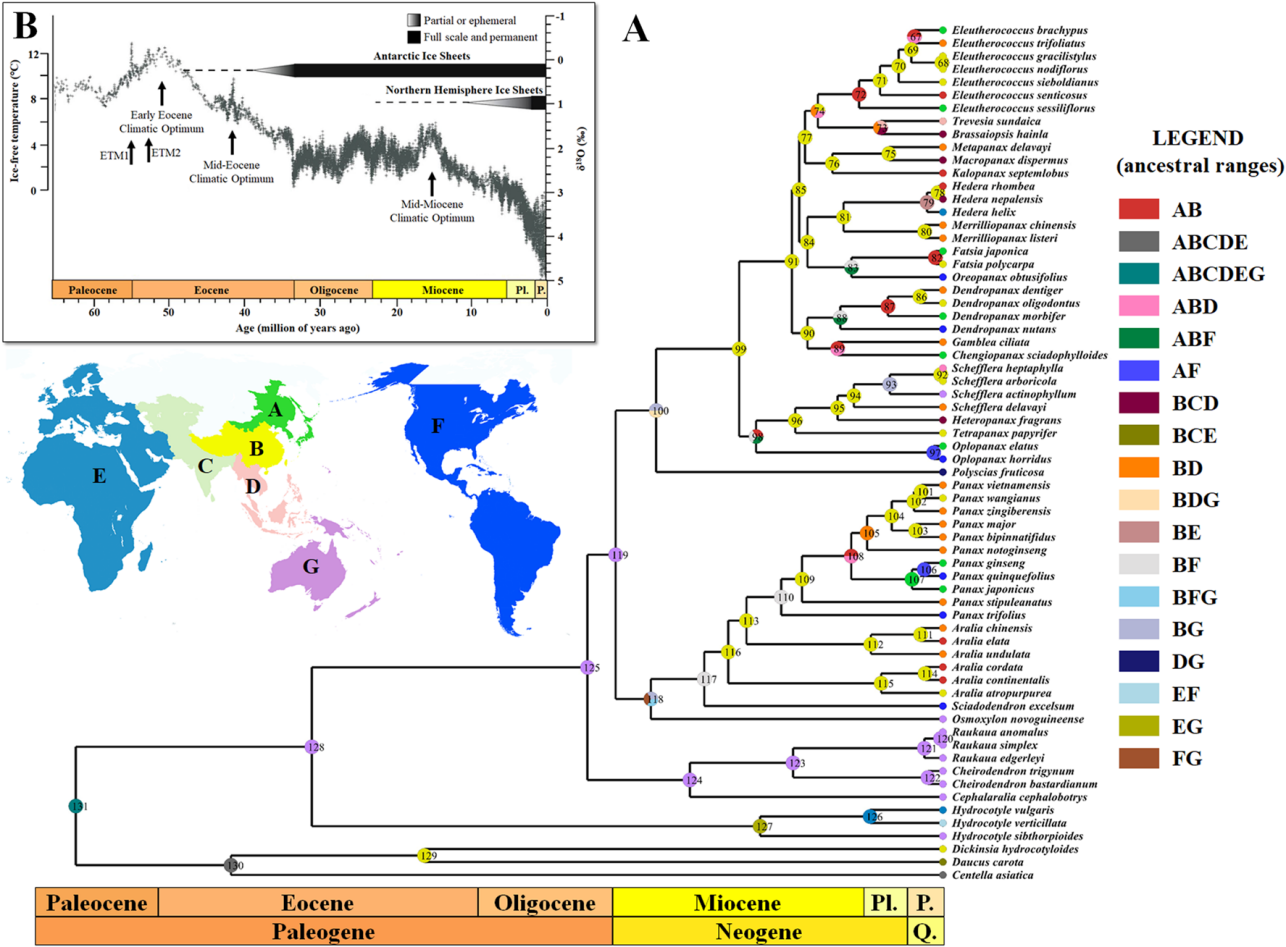
Ancestral distribution and divergence time estimation of the Araliaceae with global climate over the last 65 million years. (**A**) Graphical results of ancestral distribution areas at each node of the phylogeny of the Araliaceae obtained by S-DIVA, and divergence times for Araliaceae species were estimated by BEAST. The split between Apiaceae and Araliaceae was set as the calibration point. The map with ancestral ranges was illustrated using MapChart (https://www.mapchart.net/). Colour key indicates possible ancestral ranges; letter combinations indicate a combination of ranges. (**B**) Climate for the Cenozoic (0–65 Ma). The climatic curve is a stacked deep-sea benthic foraminiferal oxygen-isotope curve and was modified from Zachos et al.^51^. Q.: Quaternary; Pl.: Pliocene; P.: Pleistocene.

**Figure 5:**
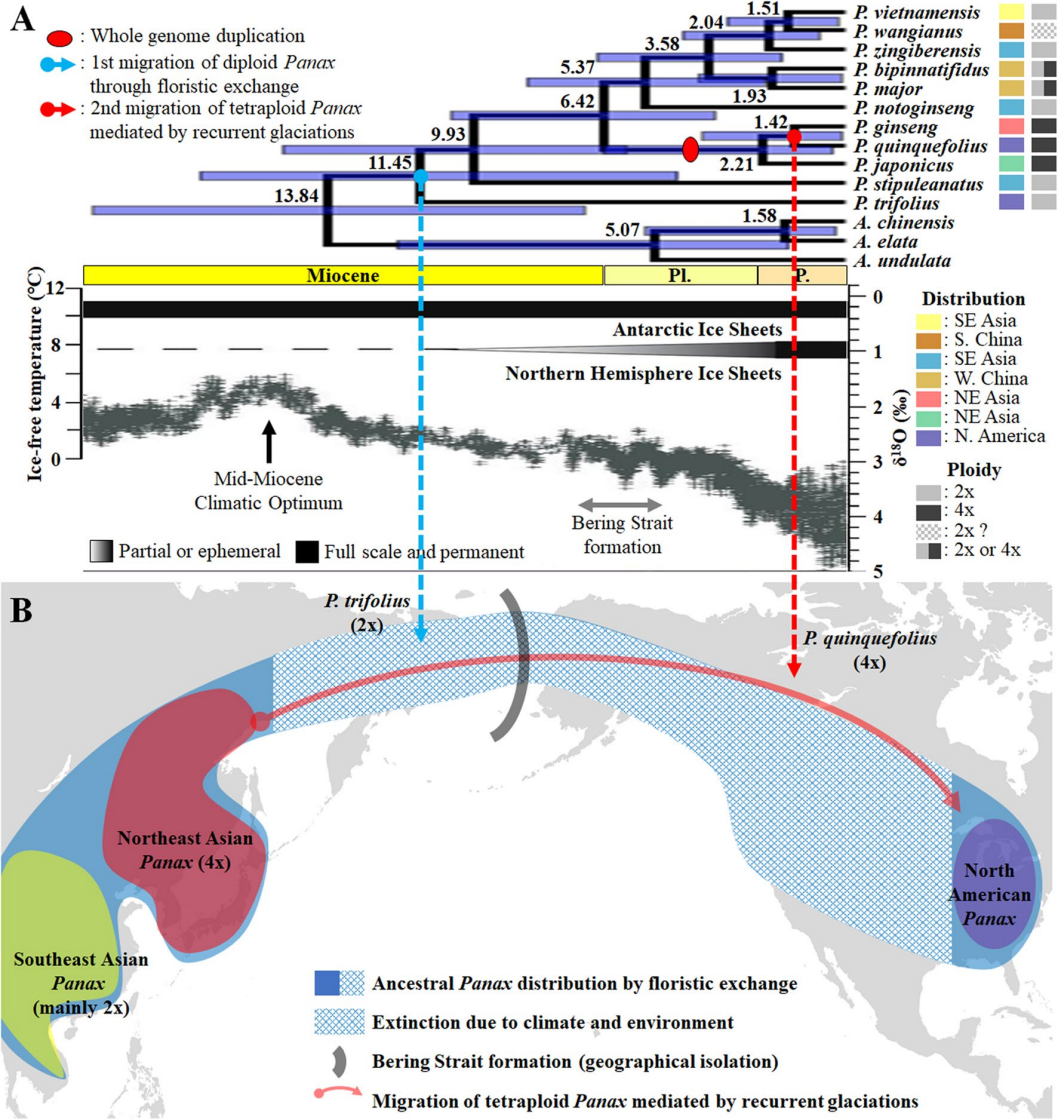
Evolutionary model of *Panax* species. (**A**) Estimated divergence times for *Panax* species with information on their distribution and ploidy level. The predicted climate for the most recent 20 million years and the curve were modified from Zachos et al.^51^. (**B**) Supplementary hypothesis involving two-stage migration of *Panax* to North America: (1) migration of ancestral diploid *Panax* through floristic exchange and (2) migration of tetraploid *Panax* mediated by recurrent glaciations. The map indicating the distribution areas of *Panax* species was downloaded from MapChart (https://www.mapchart.net/). SE: southeastern; S.: southern; NE: northeastern; W.: western; N.: north; Pl.: Pliocene; P.: Pleistocene.

We performed molecular clock analysis to estimate divergence time based on concatenated sequences of 78 plastid genes using BEAST (Supplementary Fig. S8). Our results indicated that the split within the Araliaceae began around 45 million years ago (Ma) during the Middle Eocene. The first split occurred between the two subfamilies, Aralioideae and Hydrocotyloideae, at an estimated divergence time of 44.76 Ma during the Middle Eocene (68.23–25.37 Ma in 95% HPDs). Within the Aralioideae, splits between the five major groups (*Aralia*–*Panax* group, Asian Palmate group, Greater *Raukaua* group, *Polycias–Pseudopanax* group, and *Osmoxylon* group) occurred during the Late Oligocene and the Early Miocene (Supplementary Fig. S8). During the Middle Miocene, the split of *Aralia* and *Panax* occurred at 13.84 Ma, while rapid divergence of clades showing phylogenetic discordance in the Asian Palmate group occurred from 14.36 to 9.61 Ma (Figs. 1, 2B,C, 4A, Supplementary Fig. S8). Both the divergence of the two subfamilies and the divergences within the *Aralia*–*Panax* group (the split between *Aralia* and *Panax*) and the Asian Palmate group (rapid divergence of clades showing phylogenetic discordance) were estimated to coincide with climatic optima in the Middle Eocene and the Middle Miocene (Fig. 4B). Within the genus *Panax*, two *Panax* species distributed in North America diverged from other *Panax* species at separate times. The first North American species, *P. trifolius* (dwarf ginseng), phylogenetically located in the basal group of the genus *Panax*, diverged from other *Panax* species at 11.45 Ma; the second basal species, *P. stipuleanatus*, distributed in South East Asia, diverged around 9.93 Ma during the Late Miocene (Fig. 5A, Supplementary Fig. S8). The other *Panax* species then diverged into two lineages, South Asian and Northeast Asian (Figs. 1, 5A, Supplementary Fig. S8). The split between these two lineages occurred at the boundary of the Miocene and Pliocene (6.42 Ma), and the other North American species, *P. quinquefolius* (American ginseng), diverged from *Panax ginseng* (Korean ginseng) of the Northeastern Asian lineage at 1.42 Ma (3.83–0.06 in 95% HPDs) (Fig. 5A, Supplementary Fig. S8).

The optimal ancestral reconstruction by the Statistical Dispersal-Vicariance Analysis (S-DIVA) on the BEAST tree proposed 17 vicariance events and 26 dispersal events within the Araliaceae (Fig. 4A). Ancestral area reconstruction suggested Oceania (G) as the base for the Araliaceae, which may be due to the basal *Hydrocotyle* and the Greater Raukaua groups being distributed in Oceania. After the *Hydrocotyle* divergence, the remaining Araliaceae species dispersed from Oceania (G) to Southeast Asia, the Himalayas, up to Central China (BD) (node 100) for the Asian Palmate group, and farther into South and Central Asia to the Americas (BCDF) for the *Aralia*–*Panax* group (node 118). The ancestral distribution area for both the Asian Palmate group and the *Aralia*–*Panax* group was predicted to be South and Central China (B). A total of five vicariance events were detected in the *Aralia*–*Panax* group, four of which were in the *Panax* genus (nodes 105–107, 110). Likewise, five dispersal occurrences took place in this group, with four occurring within the *Panax* genus (nodes 106, 108–110).

## Discussion

### Phylogenetic discordance and the evolution of the Asian Palmate group

Phylogenetic discordance and poly- or paraphyly of several genera have previously emerged as scientific questions in the Araliaceae^1,2,4,17–20^. Despite the use of plastome and nuclear data in recent phylogenomic studies^31,42^, these questions remain uncertain owing to the limited sampling. The phylogenetic discordance between nuclear and organelle gene trees, which is referred to as cytonuclear discordance, is known to be caused by gene paralogy, hybridization, introgression (including chloroplast capture), and incomplete lineage sorting^43–45^. However, phylogenetic discordance has often been reported in plastome-based phylogenomic studies as well. It can be caused by evolutionary rate heterogeneity between plastid genes^46^, but also by how partitioning of the plastome data was performed^47–49^. Differences in data partitioning can result in different tree topology, branch length, and bootstrap support values^50^. All the phylogenies constructed in this study supported the monophyly of the Asian Palmate group, the *Aralia*–*Panax* group, the Greater *Raukaua* group, and the genus *Hydrocotyle*, which was consistent with previous studies using few genes or genome data^1,2,4,17–20,31,42^. However, our analyses inferred different phylogenetic topologies and branch support values from four different data matrices of plastome data and the nrDNA phylogeny, particularly in the Asian Palmate group (Fig. 2). While the phylogenetic relationships within the *Aralia*–*Panax* group, the Greater *Raukaua* group, and the genus *Hydrocotyle* were consistent in all four plastome-based phylogenies (Fig. 1), those of the Asian Palmate group differed by data partitioning of plastome data and between plastome and nrDNA phylogenies as well.

Within the Asian Palmate group, phylogenetic discordance was observed in the position of four clades, the *Macropanax*–*Metapanax*–*Kalopanax* clade (Clade 2), the *Fatsia*–*Oreopanax* clade (Clade 4), the *Dendropanax*–*Chengiopanax*–*Gamblea* clade (Clade 5), and the *Oplopanax* clade (Clade 7). While these clades were monophyletic with strong branch support, phylogenetic relationships among the clades differed with the different data matrices, and their branch support values were relatively lower than those of other branches (Figs. 1, 2D). Most previous studies using few gene sequences were often failed to resolve phylogenetic relationships among groups and clades of the Asian Palmate group by showing polytomy and collapsing monophyly of them^1,2,17–19^. Our topology inferred from first and second sites of CDSs and protein sequences (Fig. 2Bii,C) was similar to the topology inferred from nrITS and six plastid regions by Li and Wen^19^, but relationships among the *Merrilliopanax*–*Hedera* clade (Clade 3), the *Fatsia*–*Oreopanax* clade (Clade 4), *Dendropanax* (Clade 5), the *Eleutherococcus*–*Brassaiopsis*–*Trevesia* clade (Clade 1), and the *Macropanax*–*Metapanax*–*Kalopanax* clade (Clade 2) remained unclear^19^. Recently, plastid and nuclear (Hyb-seq) phylogenomic studies were conducted to resolve the phylogenetic relationships of the Asian Palmate group, respectively^31,42^. The topology inferred from plastome data by Valcárcel and Wen^31^ was consistent with the topology inferred from whole-plastome sequence (Fig. 2Bi,Ci). However, the phylogenomic study by Gallego-Narbón et al.^42^ using nuclear genes indicated a novel topology that differed from any other topology inferred from plastome and nrDNA data^1,2,4,19,31^. In the topology, the *Eleutherococcus*–*Brassaiopsis*–*Trevesia* clade (Clade 1) was collapsed and divided into two places. The *Eleutherococcus* genus was nested within the *Macropanax*–*Metapanax*–*Kalopanax* clade (Clade 2), and the *Brassaiopsis*–*Trevesia* clade was sister to the *Fatsia*–*Oreopanax* clade (Clade 4)^42^. The *Oplopanax* clade (Clade 7) was sister to all other clades of the Asian Palmate group in recent studies^18,19,31,42^, and this position was supported by whole-plastome sequence in this study (Fig. 2Ci). However, different positions of the *Oplopanax* were reported, e.g., sister to the *Gamblea*^1^ and *Fatsia*^2,18^. In this study, a sister relationship between the *Schefflera*–*Heteropanax*–*Tetrapanax* clade (Clade 6) and the *Oplopanax* clade (Clade 7), inferred from 78 CDS and protein sequences, has not been reported so far, and the relationship might be an alternative hypothesis for the phylogenetic position of the *Oplopanax* clade (Clade 7).

The phylogenetic problems of the Asian Palmate group, such as incongruences between different gene tree and unsettled relationships have prompted several phylogenetic studies, and it has been hypothesized that the phylogenetic discordance might be the result of rapid diversification^18,31,42^. Rapid diversification of the Asian Palmate group was also supported by phylogenetic analyses and divergence time estimation in this study. It has recently been hypothesized that the rapid diversification of the Asian Palmate group was triggered by hybridization^42^. The limitations of maternally inherited plastome data preclude us from discussing the hypothesis proposed by Gallego-Narbón et al.^42^. Alternatively, we propose a hypothesis based on our dating results and the climatic data in the Cenzoic predicted by Zachos et al.^51^. Our molecular dating results indicated that the clades showing phylogenetic discordance in the Asian Palmate group diverged during the Middle Miocene (Fig. 4A), coinciding with the Mid-Miocene Climatic Optimum (Fig. 4B)^51^. We therefore hypothesize that the global warming event in the Middle Miocene may have triggered rapid diversification, leading to phylogenetic discordance within the Asian Palmate group. Further study using extensive sampling and reliable fossil data for more accurate molecular dating of the Asian Palmate group is needed to demonstrate how the species of the Asian Palmate group evolved.

### Accelerated AT-biased mutation in the plastomes of the semi-aquatic *Hydrocotyle* genus

Although the Araliaceae plastomes are under purifying selection (*d*_*N*_/*d*_*S*_ < 1), the accelerated substitution rates in the *Hydrocotyle* plastomes observed in this study (Supplementary Fig. S6) were also reported in the nuclear and plastid phylogenies with quite long branches, even compared with one Apiaceae species^42^. Mutational pressure and gene conversion affect the nucleotide landscape of the genome, and these two factors are known as non-adaptive mechanisms^52^. Owing to the influence of AT-biased mutational pressure in non-coding regions, the majority of land plants have AT-rich plastomes^53^; conversely, genic regions appear to be under GC-biased mutational pressure, referred to as GC-biased gene conversion^52–56^. The Araliaceae species in this study have AT-rich plastomes with a GC content of approximately 38%, suggesting that the Araliaceae plastomes are under AT-biased mutational pressure similar to other land plant plastomes. The plastomes of the subfamily Aralioideae were found to be GC-biased in the genic region, with T-to-C transitions being significantly more common than C-to-T transitions (Table 1; Supplementary Fig. S7). By contrast, the number of C-to-T transitions in *Hydrocotyle* plastomes of the subfamily Hydrocotyloideae was almost double that in Aralioideae plastomes, resulting in a slight reduction in plastome GC content (Table 1; Supplementary Fig. S7). AT-biased mutations in the *Hydrocotyle* plastomes contributed to accelerated nucleotide substitution rates and long branches in the phylogenetic trees compared with Aralioideae plastomes (Supplementary Figs. S2–S7), implying that the *Hydrocotyle* plastomes have their own evolutionary history not experienced by other species in the Araliaceae. This may be an evolutionary result of adaptation. The *Hydrocotyle* genus is the only aquatic or semi-aquatic plant in the Araliaceae family. *Hydrocotyle* species, including the three species in this study, usually grow in places that are marshy, boggy, and wet, but can even survive under water^57–59^. Accelerated nucleotide substitution rates in some species or clades exhibiting distinct environments and growth habitats have been reported^60–62^, and associated mutation rates among the plastome, mitogenome, and nuclear genome were discussed in previous studies^56,63–66^. The *Hydrocotyle* species had a relatively long branch in both plastome and nrDNA phylogenies (Fig. 3), implying that accelerated substitution rates occurred at least in plastome and nrDNA. The accelerated substitution rates in the three genome compartments may have been influenced by generation time, plant height, the replication mechanism of organelle genomes, and an improper organelle DNA repair system^56,60–66^. Therefore, we speculate that the accelerated substitution rate in the *Hydrocotyle* genus occurred not only in their plastome but also in their mitogenome and nuclear genomes as a consequence of their evolutionary history and environmental adaptation. Further research, including three genome compartments in the plant cell, may be able to reveal why *Hydrocotyle* plastomes have such a distinctive evolutionary pattern.

### Evolution of the *Aralia*–*Panax* group and two independent migrations of *Panax* species

The *Aralia*–*Panax* group consists of three genera: *Aralia*, *Panax*, and *Sciadodendron* (a monotypic genus that includes only *S. excelsum* and was recently considered *Aralia excelsa*)^17^. Two genera, *Aralia* L. and *Panax* L., include approximately 60 and 18 species, respectively, and are mainly distributed in Asia and the Americas^17,35,64^. The two genera can be distinguished by leaf and flower morphology. The *Aralia* has usually pinnate leaves and five to eight carpels, whereas the *Panax* has usually palmately compound leaves and whorled arrangements, a single terminal flower, and a bi- or tricarpellate ovary^2,17,67^. However, the phylogenetic relationships between these two genera remain uncertain so far. Previous studies using a few DNA barcoding regions showed that the *Aralia* was not monophyletic and the *Panax* was nested within the *Aralia* clade^1,2,4,17,18^. In addition, monophyly of the *Panax* was often collapsed by *P. trifolius* locating with *Aralia* species^17^, as shown in our nrDNA phylogeny (Fig. 3). In contrast, cases that supported monophyly of the two genera have also been reported^19,31,42,68^. When the *Sciadodendron* (*S. excelsum* = currently *Aralia excelsa*) was treated as an independent genus, it was sister to the *Panax*, and they were sister to the monophyletic *Aralia* clade; conversely, when it was considered a member of *Aralia*, the monophyly of the *Aralia* genus collapsed^19,31^, as shown in our plastome phylogenies (Fig. 1, 3). Two recent studies utilized a large amount of nuclear data to reconstruct the phylogenetic relationships of the Araliaceae^42,68^. Both nuclear phylogenomic studies successfully resolved and supported the monophyly of the *Panax*; however, the generic delimitation between the *Aralia* and *Panax* was still controversial^42,68^. In the two studies, *Panax* species formed their own clade with strong support^42,68^, but they were still nested within the genus *Aralia*, owing to the position of *A. humilis* (section *Humiles*)^68^. Therefore, collapsing the monophyly of the genus *Panax* is likely caused by insufficient data, but the generic delimitation of the genus *Aralia* should be revised with extensive sampling of both *Aralia* and *Panax* from Asia and the Americas, based on a large amount of nuclear data and morphology.

The *Panax* genus exhibits interesting evolutionary patterns in terms of its distribution and ploidy levels. *Panax* species are mostly distributed in East Asia and rarely in North America^35,69^. Only two *Panax* species, diploid *P. trifolius* (dwarf ginseng) and tetraploid *P. quinquefolius* (American ginseng), are distributed in the Northeastern region of North America, and their phylogenetic positions are far from each other: *P. trifolius* is sister to all *Panax* species, and *P. quinquefolius* is sister to *P. ginseng* (Korean ginseng) in our plastome phylogeny (Figs. 1, 3). The remaining East Asian *Panax* species can be divided into two groups: the diploid group distributed in Southeast Asia (Geographical area B in Fig. 4) and the tetraploid group distributed in Northeast Asia (Geographical area A in Fig. 4)^30,34,35,70^. While the Northeast Asian tetraploid *Panax* species were sister to the Southeast Asian diploid *Panax* species in the plastome phylogeny, they were located within the Southeast Asian diploid *Panax* species in the nrDNA phylogeny (Fig. 3). The cytonuclear discordance between plastome and nrDNA phylogenies could be evidence for allotetraploidization. Allotetraploidization of the Northeast Asian tetraploid *Panax* species was hypothesized previously^33^ and supported by phylogenetic evidence that the two subgenomes of the tetraploid species were located in two different clades; one subgenome (subgenome B) was sister to the *Panax notoginseng* genome and the other subgenome (subgenome A) was not^71^. These phylogenetic results imply that the allotetraploidization occurred in the common ancestor of the tetraploid *Panax* species, *P. ginseng*, *P. japonicus*, and *P. quinquefolius*, and that the migration of *P. quinquefolius* from East Asia to North America should have occurred after the allopolyploidization. Therefore, a two-step migration hypothesis has previously been proposed^33–35^, and our biogeographical analysis also supported the two independent migrations (dispersal) from East Asia to North America (Fig. 4A).

The Bering land bridge is proposed as the migration route of North American *Panax* species^33–35,72^. The first dispersal of *Panax* species (*P. trifolius*) from East Asia to North America is thought to have occurred through the Bering land bridge during the Middle Miocene (Figs. 4A, 5A,B), similar to most intercontinental disjunct plants that diverged from 3 to 25 Ma through floristic exchanges between East Asia and North America^72–74^. The Bering Strait was not formed until the Middle Miocene, with East Asia and North America instead connected by land, facilitating floristic exchanges between the two continents^74^. However, the second migration, that of tetraploid *Panax* species (*P. quinquefolius*), occurred during the Pleistocene, after the formation of the Bering Strait (Figs. 4A, 5A,B); the Bering Strait was also dynamic, experiencing recurrent glaciations and global warming^75^. This implies that natural dispersal may have been difficult owing to geographical and climatic isolation and that some mediator was needed for migration. It has previously been suggested that birds or small mammals might have carried *Panax* species into North America^76–78^. Furthermore, during the Early Pleistocene, the steppe mammoth (*Mammuthus trogontherii*), a trunked mammal, was estimated to have migrated from Eurasia to North America around 1.3–1.5 Ma^79^, coinciding with the split between *P. ginseng* and *P. quinquefolius* in this study (Figs. 4A, 5A). The migration route of the steppe mammoth reached the northeastern region of North America, where North American ginseng is currently distributed^80^. Consequently, we propose a supplementary hypothesis for the intercontinental disjunction of *Panax* species in which the North American *Panax* species migrated from East Asia to North America in two stages: the first stage was dispersal of *P. trifolius* through floristic exchange via the Bering land bridge (node 110 in Fig. 4)^72–74^, and the second stage was migration of *P. quinquefolius* (node 106 in Fig. 4), which occurred during recurrent glaciations and was likely mediated by animals, including birds and small mammals^33,35,76–78^, as well as trunked mammals.

In conclusion, our findings provide evidence that rapid diversification within the Asian Palmate group may be responsible for the phylogenetic discordance in the group, and that the *Hydrocotyle* plastome has undergone a different evolutionary history compared with other Araliaceae plastomes. In addition, we complement the hypothesis for the intercontinental disjunction of *Panax* species by combining evidence from molecular dating, distribution, and ploidy level. We believe that our study furthers our understanding of the diverse evolutionary patterns in the plastomes of the Araliaceae and complements the hypothesis for the evolution of Araliaceae species. Further studies may be able to demonstrate how the *Hydrocotyle* plastomes are under different mutational pressure from other Araliaceae plastomes as well as reveal the origin of the genus *Aralia* and whether this genus is monophyletic or evolved convergently.

## Methods

### Plant materials, DNA extraction, and Illumina sequencing

Fresh leaves of 12 species were collected from the Medicinal Plant Garden, College of Pharmacy, Seoul National University (Goyang, Korea), with permission from the garden authorities (Supplementary Table S3). Total genomic DNA was extracted using an Exgene Plant SV Midi Kit (Geneall Biotechnology, Seoul), and DNA quality was examined using gel electrophoresis and a NanoDrop 2000 spectrophotometer (Thermo Fisher Scientific, USA). Library preparation and sequencing were performed by Phyzen Co. Ltd. (Seongnam, South Korea). Approximately 1 μg of genomic DNA for each sample was sequenced using two NGS platforms. Of the 12 newly sequenced species, seven were sequenced using the Illumina MiSeq platform with paired-end reads of 2 × 300 bp and and five were sequenced using the Illumina Hiseq Xten with paired-end reads of 2 × 150 bp (Supplementary Table S1).

### Plastome and nrDNA assembly and gene annotation

In addition to 12 newly sequenced species, raw reads of *Hydrocotyle vulgaris* (ERX5309978) were downloaded from the NCBI Sequence Read Archive (SRA) database for plastome and 45S nrDNA assemblies. Plastid genome and 45S nrDNA sequences were assembled using the dnaLCW method^40,41^. Sequence trimming and de novo assembly of plastid genomes and 45S rDNA were performed using the CLC assembly cell v. 4.21 (CLC Bio, Denmark). Plastome (KM088019) and 45S rDNA (KM036295) sequences of *Panax ginseng* cultivar ‘Chunpoong’ were used as a reference. Contigs belonging to the plastome were extracted using MUMmer^81^ and BLASTZ^82^, and assembled contigs were curated manually. Gene annotation of the plastid genome was performed using GeSeq^83^ and Artemis^84^. For the 45S nrDNA contig, annotation of 18S, ITS-1, 5.8S, ITS2, and 26S was determined through sequence comparison with the reference. Circular maps of plastome structures were drawn using Circos^85^.

### Phylogenetic reconstruction

To reconstruct the plastome-based phylogeny of the Araliaceae, 53 previously reported plastome sequences, including 10 plastome sequences reported by the authors’ laboratory, were downloaded from NCBI GenBank (Supplementary Table S3). These 53 sequences plus the 13 plastomes newly assembled in this study (66 plastome sequences including three Apiaceae species as the outgroup) were used to reconstruct the phylogenetic relationships of the Araliaceae (Supplementary Table S3). Phylogenetic reconstruction was performed using four different matrices: (1) whole-plastome sequences, (2) concatenation of 78 protein-coding gene sequences, (3) first and second sites of 78 coding sequences (CDSs), and (4) protein sequences of 78 genes. Sequence alignments for whole-plastome sequences and for each gene were conducted using MAFFT^86^, and the aligned sequences of each gene were then concatenated. For phylogenetic reconstruction using protein sequences, the CDS of each gene was translated into an amino acid sequence using the translator embedded in BioEdit v. 7.2.5^87^; amino acid sequences were then aligned using MAFFT. Phylogenetic analyses based on the four different matrices were conducted using RAxML v. 8^88^ with 1000 bootstrap replicates and models selected by jModeltest v. 2.1.5^89^. The nrDNA sequences of 25 species (including *Centella asiatica* as an outgroup) were used to reconstruct a phylogenetic tree of 45S nrDNA sequences, including 18S, 5.8S, and 26S rDNAs and two ITS regions. Alignment and phylogenetic reconstruction were performed using MAFFT and RAxML, respectively. In order to compare phylogenetic results between nrDNA and plastome phylogenies, a plastome-based phylogeny using 78 protein-coding gene sequences of these 25 species was reconstructed according to the alignment and phylogenetic analysis described above.

### Nucleotide substitution analyses

Synonymous (*d*_*S*_) and nonsynonymous (*d*_*N*_) substitution rates of 78 plastid genes were estimated using the codeml application in PAML v. 4.9^90^. The topology obtained from phylogenetic reconstruction was used for nucleotide substitution rate analysis. Plastid gene sequences of *Centella asiatica* (Apiaceae) were included as a standard, and the F3 × 4 model was used for codon frequencies. Statistical significances between different groups were examined using analysis of variance (ANOVA) with Bonferroni post hoc tests using R v. 3.5.1^91^. To determine the presence of any specific mutational pressure in the genomes, substitution types in protein-coding regions, such as C-to-T or T-to-C transitions and transversions between purine and pyrimidine bases were investigated.

### Divergence time estimation

Bayesian divergence time estimation was performed using a lognormal relaxed clock model in BEAST 2 v. 2.6.2^92^. A total of 66 species were included, and 78 concatenated plastid gene sequences (excluding *rps12* and *ycf15* genes) were used. The GTR model with invariant sites and a number of six gamma categories was selected as the best-fit model. One secondary nodal calibration was employed, with the root age of Apiaceae and Araliaceae constrained using a normal prior distribution with mean of 60.19 and SD of 6.6, according to the results of Magallon et al.^93^. The dataset was run using a birth–death speciation prior, and 78 independent runs of 10 million generations with sampling every 1000 generations were performed. The log files obtained from independent runs were combined using LogCombiner in BEAST after burning the first 10% of generations; the combined logs were then checked using Tracer v. 1.7.2^94^ to confirm whether effective sample sizes for relevant parameters were over 200. Tree files were then resampled using LogCombiner with the same burn-in strategy, and a time-calibrated maximum clade credibility (MCC) tree with 0.5 posterior probability limit and mean branch lengths was generated using TreeAnnotator in BEAST. The estimated divergence time tree was displayed using FigTree v.1.4.3 to check mean and 95% high posterior densities (HPDs) of the chronogram.

### Ancestral area estimation

Biogeographical analysis was performed with the S-DIVA to reconstruct the possible ancestral ranges of the Araliaceae on the phylogenetic tree, implemented in the Reconstruct Ancestral State in Phylogenies (RASP) v. 4.3^95^. A total of 66 species were assigned to seven geographical regions according to their native distribution provided by Plants of the World Online (https://powo.science.kew.org/): (A) Northeast Asia, (B) Southeast China, Central China, Himalayas, (C) Central Asia, Southern Asia, (D) Southeast Asia, (E) Europe, West Asia, Africa, (F) North, Central, South America, (G) Australia, Oceania, Pacific Islands. The seven geographical regions were color-coded and illustrated using MapChart (https://www.mapchart.net/). Maximum areas at each node were set to seven. The S-DIVA analysis was run allowing for extinction.

### Sample collection and experiment statement

All the plant materials in this study were collected with permission from the garden authorities, and all the methods in this study comply with relevant institutional, national, and international guidelines and legislation.

### Supplementary Information

Supplementary Figures.

Supplementary Tables.

## Data Availability

All relevant data can be found within the manuscript and its supporting materials. Newly assembled plastid genome and 45S nrDNA sequences of 12 species have been deposited in GenBank, and the accession numbers are included within the Supplementary Information. Other data and materials provided in this manuscript are available from the corresponding author upon reasonable request.

## References

[1] Wen J, Plunkett GM, Mitchell AD, Wagstaff SJ (2001). The evolution of Araliaceae: A phylogenetic analysis based on ITS sequences of nuclear ribosomal DNA. Syst. Bot..

[2] Plunkett GM, Wen J, Lowry PP (2004). Infrafamilial classifications and characters in Araliaceae: Insights from the phylogenetic analysis of nuclear (ITS) and plastid (*trn*L-*trn*F) sequence data. Plant Syst. Evol..

[3] Nuraliev MS, Oskolski AA, Sokoloff DD, Remizowa MV (2010). Flowers of Araliaceae: Structural diversity, developmental and evolutionary aspects. Plant Div. Evol..

[4] Mitchell A, Li R, Brown JW, Schönberger I, Wen J (2012). Ancient divergence and biogeography of *Raukaua* (Araliaceae) and close relatives in the southern hemisphere. Aust. Syst. Bot..

[5] Liu Z, Zeng X, Yang D, Chu G, Yuan Z, Chen S (2012). Applying DNA barcodes for identification of plant species in the family Araliaceae. Gene.

[6] Calestani V (1905). Contributo alla sistematica: Ombrellifere D'Europa. Webbia.

[7] Thorne, R. F. Inclusion of the Apiaceae (Umbelliferae) in the Araliaceae. Edinb. *Roy. Bot. Gard. Notes.* (1973).

[8] Judd WS, Sanders RW, Donoghue MJ (1994). Angiosperm family pairs: Preliminary phylogenetic analyses. Harv. Pap. Bot..

[9] Xiang QB, Lowry PP, Wu ZY, Raven P (2007). Araliaceae. Flora of China.

[10] Wan JB, Li SP, Chen JM, Wang YT (2007). Chemical characteristics of three medicinal plants of the *Panax* genus determined by HPLC-ELSD. J. Sep. Sci..

[11] Choi KT (2008). Botanical characteristics, pharmacological effects and medicinal components of Korean *Panax ginseng* CA Meyer. Acta Pharmacol. Sin..

[12] Hyun TK, Kim JS (2009). The pharmacology and clinical properties of *Kalopanax pictus*. J. Med. Plants Res.

[13] Sun YL, Liu LD, Hong SK (2011). *Eleutherococcus senticosus* as a crude medicine: Review of biological and pharmacological effects. J. Med. Plants Res..

[14] Zhao S, Huang Z, Gao J (2011). Lupane-type triterpenoids from the leaves of *Heteropanax fragrans*. Bull. Korean Chem. Soc..

[15] Jin KS, Oh YN, Lee JY, Son BY, Choi W, Lee EW (2013). Anti-oxidative and anti-inflammatory activities of seven medicinal herbs including *Tetrapanax papyriferus* and *Piper longum* Linne. Microbiol. Biotechnol. Lett..

[16] Zhang L, Xu Q, Zhan D, Zhang H, Xia G, Zhu J, Zang H (2020). Chemical composition and biological activities of essential oil from roots of *Aralia continentalis*. Chem. Nat. Compd..

[17] Wen J (2001). Evolution of the *Aralia*-*Panax* complex (Araliaceae) as inferred from nuclear ribosomal ITS sequences. Edinb. J. Bot..

[18] Valcárcel V, Fiz-Palacios O, Wen J (2014). The origin of the early differentiation of Ivies (*Hedera* L.) and the radiation of the Asian Palmate group (Araliaceae). Mol. Phylogenet. Evol..

[19] Li R, Wen J (2016). Phylogeny and diversification of Chinese Araliaceae based on nuclear and plastid DNA sequence data. J. Syst. Evol..

[20] Perkins AJ (2019). Molecular phylogenetics and species delimitation in annual species of *Hydrocotyle* (Araliaceae) from South Western Australia. Mol. Phylogenet. Evol..

[21] Palmer JD, Vasil LK, Bogorad L (1991). Plastid chromosomes: Structure and evolution. The Molecular Biology of Plastids.

[22] Downie SR, Palmer JD, Soltis S, Soltis DE, Doyle JJ (1992). Use of chloroplast DNA rearrangements in reconstructing plant phylogeny. Molecular Systematics of Plants.

[23] Zhang SD, Jin JJ, Chen SY, Chase MW, Soltis DE, Li HT (2017). Diversification of Rosaceae since the Late Cretaceous based on plastid phylogenomics. N. Phytol..

[24] Wei R, Yan YH, Harris AJ, Kang JS, Shen H, Xiang QP, Zhang XC (2017). Plastid phylogenomics resolve deep relationships among eupolypod II ferns with rapid radiation and rate heterogeneity. Genome Biol. Evol..

[25] Song Y, Yu WB, Tan YH, Jin JJ, Wang B, Yang JB (2020). Plastid phylogenomics improve phylogenetic resolution in the Lauraceae. J. Syst. Evol..

[26] Li HT, Luo Y, Gan L, Ma PF, Gao LM, Yang JB (2021). Plastid phylogenomic insights into relationships of all flowering plant families. BMC Biol..

[27] Serna-Sánchez MA, Pérez-Escobar OA, Bogarín D, Torres-Jimenez MF, Alvarez-Yela AC, Arcila-Galvis JE (2021). Plastid phylogenomics resolves ambiguous relationships within the orchid family and provides a solid timeframe for biogeography and macroevolution. Sci. Rep..

[28] Li R, Ma PF, Wen J, Yi TS (2013). Complete sequencing of five Araliaceae chloroplast genomes and the phylogenetic implications. PloS ONE.

[29] Kim K, Nguyen VB, Dong J, Wang Y, Park JY, Lee SC, Yang TJ (2017). Evolution of the Araliaceae family inferred from complete chloroplast genomes and 45S nrDNAs of 10 *Panax*-related species. Sci. Rep..

[30] Ji Y, Liu C, Yang Z, Yang L, He Z, Wang H (2019). Testing and using complete plastomes and ribosomal DNA sequences as the next generation DNA barcodes in *Panax* (Araliaceae). Mol. Ecol. Resour..

[31] Valcárcel V, Wen J (2019). Chloroplast phylogenomic data support Eocene amphi-Pacific early radiation for the Asian Palmate core Araliaceae. J. Syst. Evol..

[32] Choi HI, Kim NH, Lee J, Choi BS, Kim KD, Park JY (2013). Evolutionary relationship of *Panax ginseng* and *P. quinquefolius* inferred from sequencing and comparative analysis of expressed sequence tags. Genet. Resour. Crop Evol..

[33] Kim NH, Jayakodi M, Lee SC, Choi BS, Jang W, Lee J (2018). Genome and evolution of the shade-requiring medicinal herb *Panax ginseng*. Plant Biotechnol. J..

[34] Zuo YJ, Wen J, Zhou SL (2017). Intercontinental and intracontinental biogeography of the eastern Asian–eastern North American disjunct *Panax* (the ginseng genus, Araliaceae), emphasizing its diversification processes in eastern Asia. Mol. Phylogenet. Evolut..

[35] Shim H, Waminal NE, Kim HH, Yang TJ (2021). Dynamic evolution of *Panax* species. Genes Genom..

[36] Kovarik A, Matyasek R, Lim KY, Skalická K, Koukalova B, Knapp S (2004). Concerted evolution of 18–5.8–26S rDNA repeats in Nicotiana allotetraploids. Biol. J. Linn. Soc..

[37] Ganley AR, Kobayashi T (2007). Highly efficient concerted evolution in the ribosomal DNA repeats: Total rDNA repeat variation revealed by whole-genome shotgun sequence data. Genome Res..

[38] Álvarez IJFW, Wendel JF (2003). Ribosomal ITS sequences and plant phylogenetic inference. Mol. Phylogenet. Evol..

[39] Rodnina MV, Beringer M, Wintermeyer W (2007). How ribosomes make peptide bonds. Trends Biochem. Sci..

[40] Kim K, Lee SC, Lee J, Yu Y, Yang K, Choi BS (2015). Complete chloroplast and ribosomal sequences for 30 accessions elucidate evolution of *Oryza* AA genome species. Sci. Rep..

[41] Kim K, Lee SC, Lee J, Lee HO, Joh HJ, Kim NH (2015). Comprehensive survey of genetic diversity in chloroplast genomes and 45S nrDNAs within *Panax ginseng* species. PLoS One.

[42] Gallego-Narbón A, Wen J, Liu J, Valcárcel V (2022). Hybridization and genome duplication for early evolutionary success in the Asian Palmate group of Araliaceae. J. Syst. Evol..

[43] Maddison WP (1997). Gene trees in species trees. Syst. Biol..

[44] Folk RA, Soltis PS, Soltis DE, Guralnick R (2018). New prospects in the detection and comparative analysis of hybridization in the tree of life. Am. J. Bot..

[45] Yang YY, Qu XJ, Zhang R, Stull GW, Yi TS (2021). Plastid phylogenomic analyses of Fagales reveal signatures of conflict and ancient chloroplast capture. Mol. Phylogenet. Evol..

[46] Zhang X, Sun Y, Landis JB, Lv Z, Shen J, Zhang H (2020). Plastome phylogenomic study of Gentianeae (Gentianaceae): Widespread gene tree discordance and its association with evolutionary rate heterogeneity of plastid genes. BMC Plant Biol..

[47] Zhang HR, Wei R, Xiang QP, Zhang XC (2020). Plastome-based phylogenomics resolves the placement of the *sanguinolenta* group in the spikemoss of lycophyte (*Selaginellaceae*). Mol. Phylogenet. Evol..

[48] Du XY, Lu JM, Li DZ (2020). Extreme plastid RNA editing may confound phylogenetic reconstruction: A case study of *Selaginella* (lycophytes). Plant Divers..

[49] Lee HR, Kim KA, Kim BY, Park YJ, Lee YB, Cheon KS (2022). The complete chloroplast genome sequences of eight *Orostachys* species: Comparative analysis and assessment of phylogenetic relationships. Plos One.

[50] Kainer D, Lanfear R (2015). The effects of partitioning on phylogenetic inference. Mol. Biol. Evolut..

[51] Zachos JC, Dickens GR, Zeebe RE (2008). An early Cenozoic perspective on greenhouse warming and carbon-cycle dynamics. Nature.

[52] Smith DR (2012). Updating our view of organelle genome nucleotide landscape. Front. Genet..

[53] Smith DR, Keeling PJ (2015). Mitochondrial and plastid genome architecture: Reoccurring themes, but significant differences at the extremes. Proc. Natl. Acad. Sci..

[54] Wu CS, Chaw SM (2015). Evolutionary stasis in cycad plastomes and the first case of plastome GC-biased gene conversion. Genome Biol. Evol..

[55] Niu Z, Xue Q, Wang H, Xie X, Zhu S, Liu W, Ding X (2017). Mutational biases and GC-biased gene conversion affect GC content in the plastomes of *Dendrobium* genus. Int. J. Mol. Sci..

[56] Kang JS, Yu J, Zhang XC, Xiang QP (2022). The associated evolution among the extensive RNA editing GC-biased mutation, and PPR family expansion in the organelle genomes of *Selaginellaceae*. J. Syst. Evol..

[57] Nicolas AN, Plunkett GM (2009). The demise of subfamily Hydrocotyloideae (Apiaceae) and the re-alignment of its genera across the entire order Apiales. Mol. Phylogenet. Evol..

[58] Van De Wiel CCM, Van Der Schoot J, Van Valkenburg JLCH, Duistermaat H, Smulders MJM (2009). DNA barcoding discriminates the noxious invasive plant species, floating pennywort (*Hydrocotyle ranunculoides* Lf), from non-invasive relatives. Mol. Ecol. Resour..

[59] Wan JZ, Wang MZ, Qin TJ, Bu XQ, Li HL, Yu FH (2019). Spatial environmental heterogeneity may drive functional trait variation in *Hydrocotyle vulgaris* (Araliaceae), an invasive aquatic plant. Aquat. Biol..

[60] Schwarz EN, Ruhlman TA, Weng ML, Khiyami MA, Sabir JSM, Hajarah NH, Alharbi NS, Rabah SO, Jansen RK (2017). Plastome-wide nucleotide substitution rates reveal accelerated rates in Papilionoideae and correlations with genome features across legume subfamilies. J. Mol. Evol..

[61] Bedoya AM, Ruhfel BR, Philbrick CT, Madriñán S, Bove CP, Mesterházy A, Olmstead RG (2019). Plastid genomes of five species of riverweeds (Podostemaceae): Structural organization and comparative analysis in Malpighiales. Front. Plant Sci..

[62] Lee C, Ruhlman TA, Jansen RK (2023). Rate accelerations in plastid and mitochondrial genomes of Cyperaceae occur in the same clades. Mol. Phylogenet. Evol..

[63] Bromham L, Hua X, Lanfear R, Cowman PF (2015). Exploring the relationships between mutation rates, life history, genome size, environment, and species richness in flowering plants. Am. Nat..

[64] Kang JS, Zhang HR, Wang YR, Liang SQ, Mao ZY, Zhang XC, Xiang QP (2020). Distinctive evolutionary pattern of organelle genomes linked to the nuclear genome in *Selaginellaceae*. Plant Journal.

[65] Forsythe ES, Williams AM, Sloan DB (2021). Genome-wide signatures of plastid-nuclear coevolution point to repeated perturbations of plastid proteostasis systems across angiosperms. Plant Cell.

[66] Qu XJ, Zhang XJ, Cao DL, Guo XX, Mower JP, Fan SJ (2022). Plastid and mitochondrial phylogenomics reveal correlated substitution rate variation in *Koenigia* (Polygonoideae, Polygonaceae) and a reduced plastome for *Koenigia delicatula* including loss of all *ndh* genes. Mol. Phylogenet. Evol..

[67] Wen J (1993). Generic delimitation of *Aralia* (Araliaceae). Brittonia.

[68] Liu J, Nie ZL, Ren C, Su C, Wen J (2023). Phylogenomics of Aralia sect. *Aralia* (Araliaceae): Signals of hybridization and insights into its species delimitations and intercontinental biogeography. Mol. Phylogenet. Evol..

[69] Lee C, Wen J (2004). Phylogeny of *Panax* using chloroplast *trnC*–*trnD* intergenic region and the utility of *trnC*–*trnD* in interspecific studies of plants. Mol. Phylogenet. Evol..

[70] Shi FX, Li MR, Li YL, Jiang P, Zhang C, Pan YZ (2015). The impacts of polyploidy, geographic and ecological isolations on the diversification of *Panax* (Araliaceae). BMC Plant Biol..

[71] Wang ZH, Wang XF, Lu T, Li MR, Jiang P, Zhao J (2022). Reshuffling of the ancestral core-eudicot genome shaped chromatin topology and epigenetic modification in *Panax*. Nat. Commun..

[72] Wen J, Ickert-Bond S, Nie ZL, Li R, Long M, Gu H, Zhou Z (2010). Timing and modes of evolution of eastern Asian-North American biogeographic disjunctions in seed plants. Darwin’s Heritage Today: Proceedings of the Darwin.

[73] Wen J (1999). Evolution of eastern Asian and eastern North American disjunct distributions in flowering plants. Ann. Rev. Ecol. Syst..

[74] Wen J, Nie ZL, Ickert-Bond SM (2016). Intercontinental disjunctions between eastern Asia and western North America in vascular plants highlight the biogeographic importance of the Bering land bridge from late Cretaceous to Neogene. J. Syst. Evol..

[75] Detlef H, Belt ST, Sosdian SM, Smik L, Lear CH, Hall IR (2018). Sea ice dynamics across the Mid-Pleistocene transition in the Bering Sea. Nat. Commun..

[76] Van der Voort, M. E. *An ecological and demographic study of American ginseng (Panax quinquefolius L.) in central Appalachia*. Dissertation/Ph.D. thesis]. [Morgantown (WV), (West Virginia University, 2005).

[77] Hruska AM, Souther S, Mcgraw JB (2014). Songbird dispersal of American ginseng (*Panax quinquefolius*). Ecoscience.

[78] Elza MC, Slover C, McGraw JB (2016). Analysis of wood thrush (*Hylocichla mustelina*) movement patterns to explain the spatial structure of American ginseng (*Panax quinquefolius*) populations. Ecol. Res..

[79] Lister AM, Sher AV (2015). Evolution and dispersal of mammoths across the Northern Hemisphere. Science.

[80] Enk J, Devault A, Widga C, Saunders J, Szpak P, Southon J (2016). *Mammuthus* population dynamics in late Pleistocene North America: Divergence, phylogeography, and introgression. Front. Ecol. Evol..

[81] Kurtz S, Phillippy A, Delcher AL, Smoot M, Shumway M, Antonescu C (2004). Versatile and open software for comparing large genomes. Genome Biol..

[82] Schwartz S, Kent WJ, Smit A, Zhang Z, Baertsch R, Hardison RC (2003). Human–mouse alignments with BLASTZ. Genome Res..

[83] Tillich M, Lehwark P, Pellizzer T, Ulbricht-Jones ES, Fischer A, Bock R (2017). GeSeq–versatile and accurate annotation of organelle genomes. Nucleic Acids Res..

[84] Carver T, Harris SR, Berriman M, Parkhill J, McQuillan JA (2012). Artemis: An integrated platform for visualization and analysis of high-throughput sequence-based experimental data. Bioinformatics.

[85] Krzywinski M, Schein J, Birol I, Connors J, Gascoyne R, Horsman D (2009). Circos: An information aesthetic for comparative genomics. Genome Res..

[86] Katoh K, Standley DM (2013). MAFFT multiple sequence alignment software version 7: Improvements in performance and usability. Mol. Biol. Evol..

[87] Hall TA (1999). BioEdit: A user-friendly biological sequence alignment editor and analysis program for Windows 95/98/NT. Nucl. Acids. Symp. Ser..

[88] Stamatakis A (2006). RAxML-VI-HPC: Maximum likelihood-based phylogenetic analyses with thousands of taxa and mixed models. Bioinformatics.

[89] Darriba D, Taboada GL, Doallo R, Posada D (2012). jModelTest 2: More models, new heuristics and parallel computing. Nat. Methods.

[90] Yang Z (2007). PAML 4: Phylogenetic analysis by maximum likelihood. Mol. Biol. Evol..

[91] R Development Core Team. R*: A Language and Environment for Statistical Computing. Vienna: R Foundation for Statistical Computing*. Accessed 1 September 1, 2022, http://www.R-project.org/. (2015).

[92] Bouckaert R, Vaughan TG, Barido-Sottani J, Duchêne S, Fourment M, Gavryushkina A (2019). BEAST 2.5: An advanced software platform for Bayesian evolutionary analysis. PLOS Comput. Biol..

[93] Magallón S, Gómez-Acevedo S, Sánchez-Reyes LL, Hernández-Hernández T (2015). A metacalibrated time-tree documents the early rise of flowering plant phylogenetic diversity. N. Phytol..

[94] Rambaut A, Drummond AJ, Xie D, Baele G, Suchard MA (2018). Posterior summarization in Bayesian phylogenetics using Tracer 1.7. Syst. Biol..

[95] Yu Y, Blair C, He X (2020). RASP 4: Ancestral state reconstruction tool for multiple genes and characters. Mol. Biol. Evol..

