# Supplementary material for: Evolution of the Araliaceae family involved rapid diversification of the Asian Palmate group and Hydrocotyle specific mutational pressure
Source: Sci Rep. 2023 Dec 15;13:22325. doi: 10.1038/s41598-023-49830-7 (PMC10724125; doi:10.1038/s41598-023-49830-7)
Supplement: Supplementary file 1 — Supplementary Figures. [file 41598_2023_49830_MOESM1_ESM.pdf]

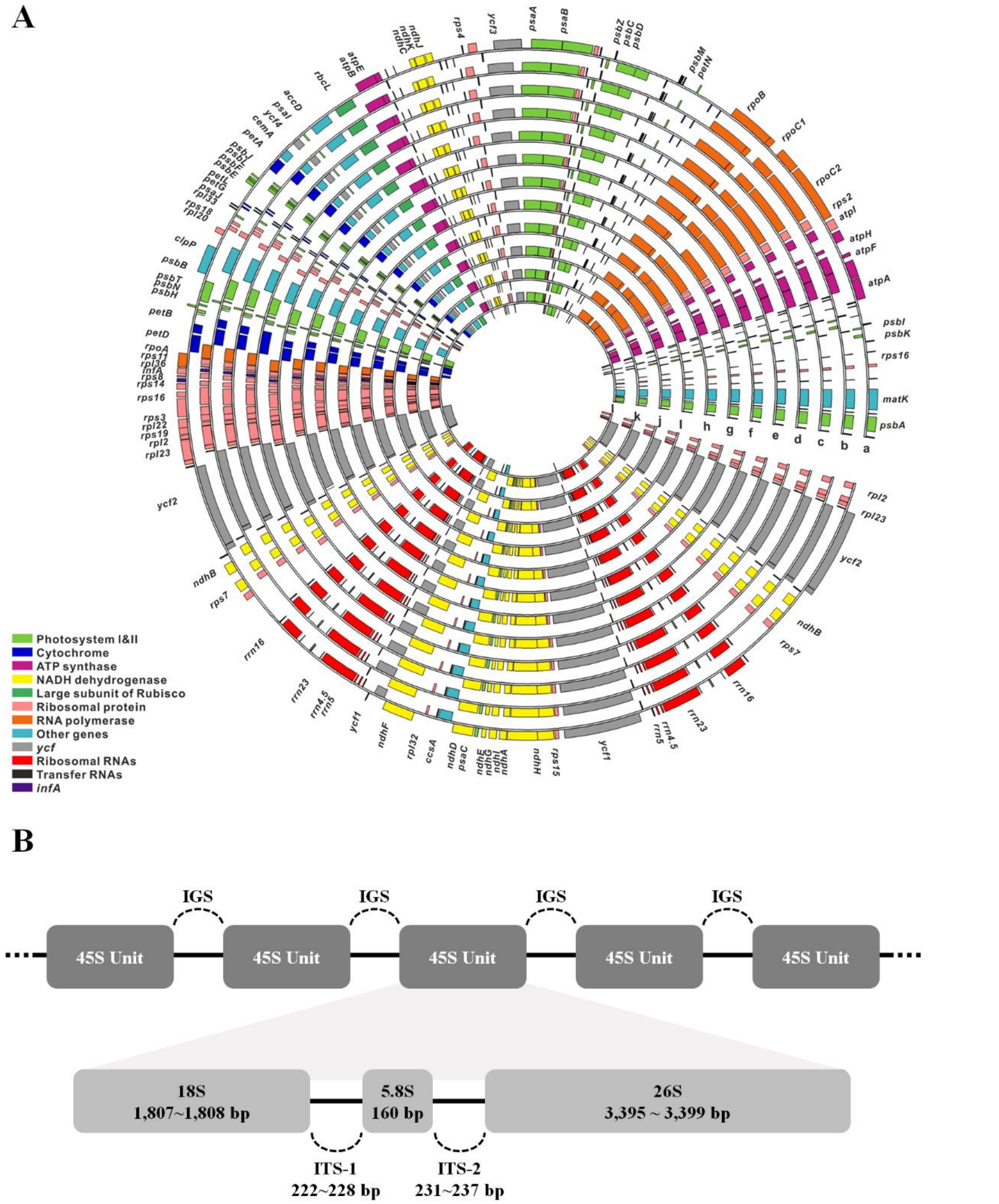

Supplementary Fig. S1. Plastid genome map and 45S nrDNA structure of 11 Araliaceae species and one Apiaceae species. (A) Circular map drawn using Circos. a: *Centella asiatica*; b: *Aralia cordata*; c: *Aralia elata*; d: *Dendropanax morbifer*; e: *Fatsia japonica*; f: *Hedera helix*; g: *Hedera rhombea*; h: *Kalopanax septemlobus*; i: *Oplopanax elatus*; j: *Polyscias fruticose*; k: *Schefflera arboricola*; l: *Tetrapanax papyrifer*. (B) Arrangement of tandemly repeated 45S nrDNA units in the nuclear genome and their subunits and length. Detailed lengths of 18S, 5.8S, 26S, and two internal transcribed spacers for each species are given in Table S2. IGS: intergenic spacer; ITS: internal transcribed spacer.

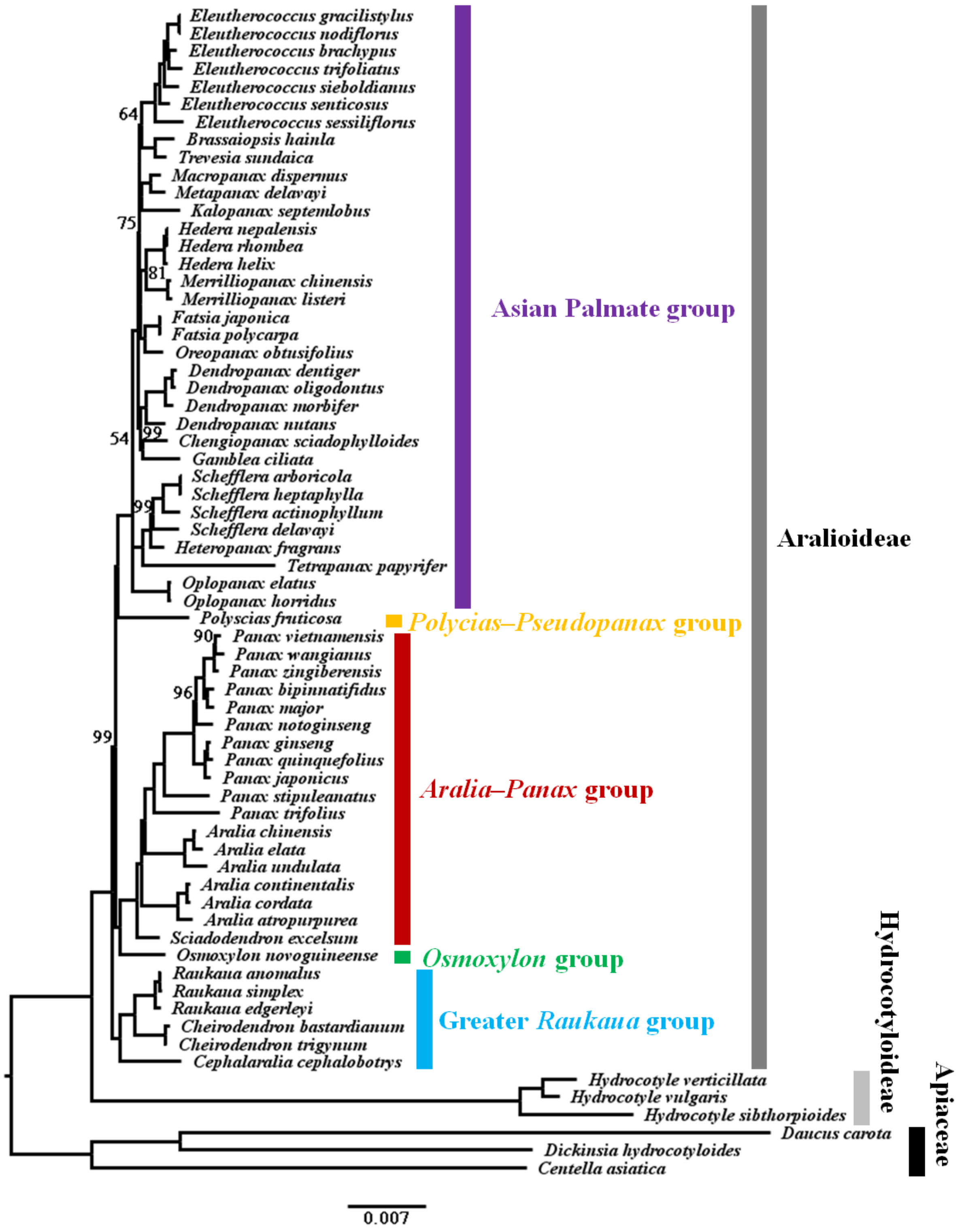

Supplementary Fig. S2. Phylogram of the best maximum-likelihood tree based on whole-plastome sequences of 63 Araliaceae species, inferred using RAxML. Three Apiaceae plastome sequences were included as an outgroup (Supplementary Table S1). Bootstrap values greater than 50% are shown near the clades.

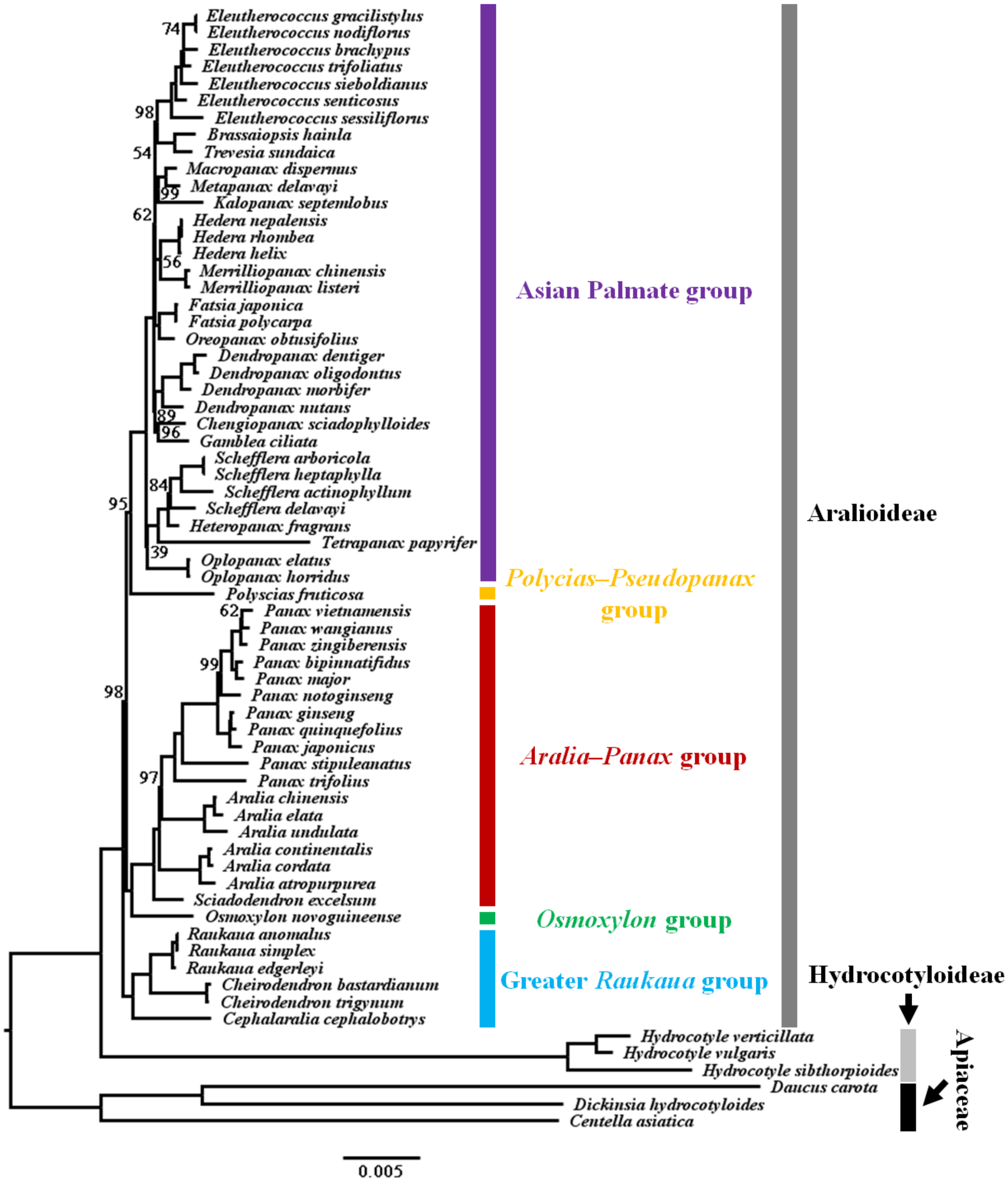

Supplementary Fig. S3. Phylogram of the best maximum-likelihood tree based on 78 protein-coding gene sequences of 63 Araliaceae species, inferred using RAxML. Three Apiaceae plastome sequences were included as an outgroup (Supplementary Table S1). Bootstrap values greater than 50% are shown near the clades.

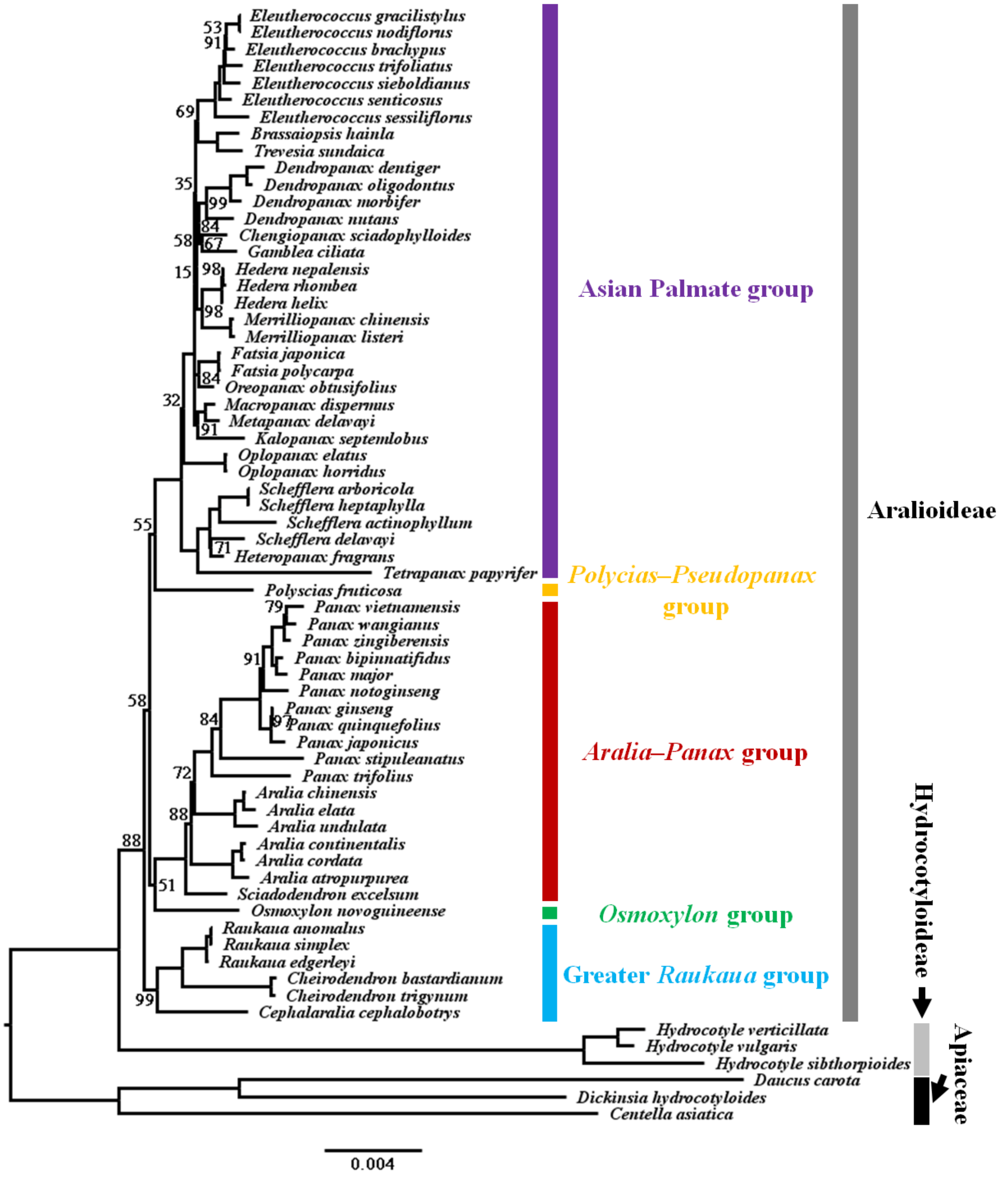

Supplementary Fig. S4. Phylogram of the best maximum-likelihood tree based on the first and second sites of protein-coding gene sequences of 63 Araliaceae species, inferred using RAxML. Three Apiaceae plastome sequences were included as an outgroup (Supplementary Table S1). Bootstrap values greater than 50% are shown near the clades.

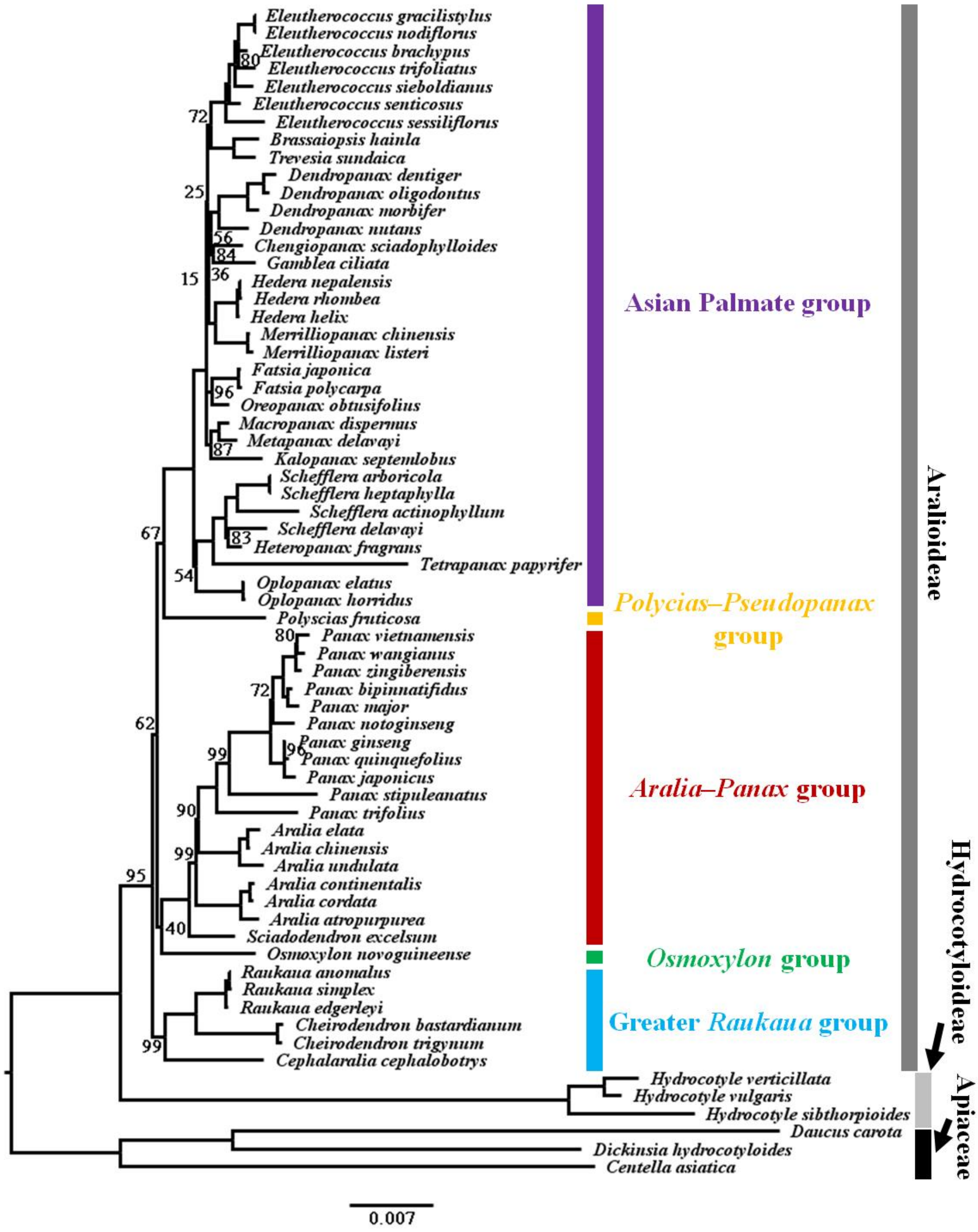

Supplementary Fig. S5. Phylogram of the best maximum-likelihood tree based on protein (amino acids) sequences of 63 Araliaceae species, inferred using RAxML. Three Apiaceae plastome sequences were included as an outgroup (Supplementary Table S1). Bootstrap values greater than 50% are shown near the clades.

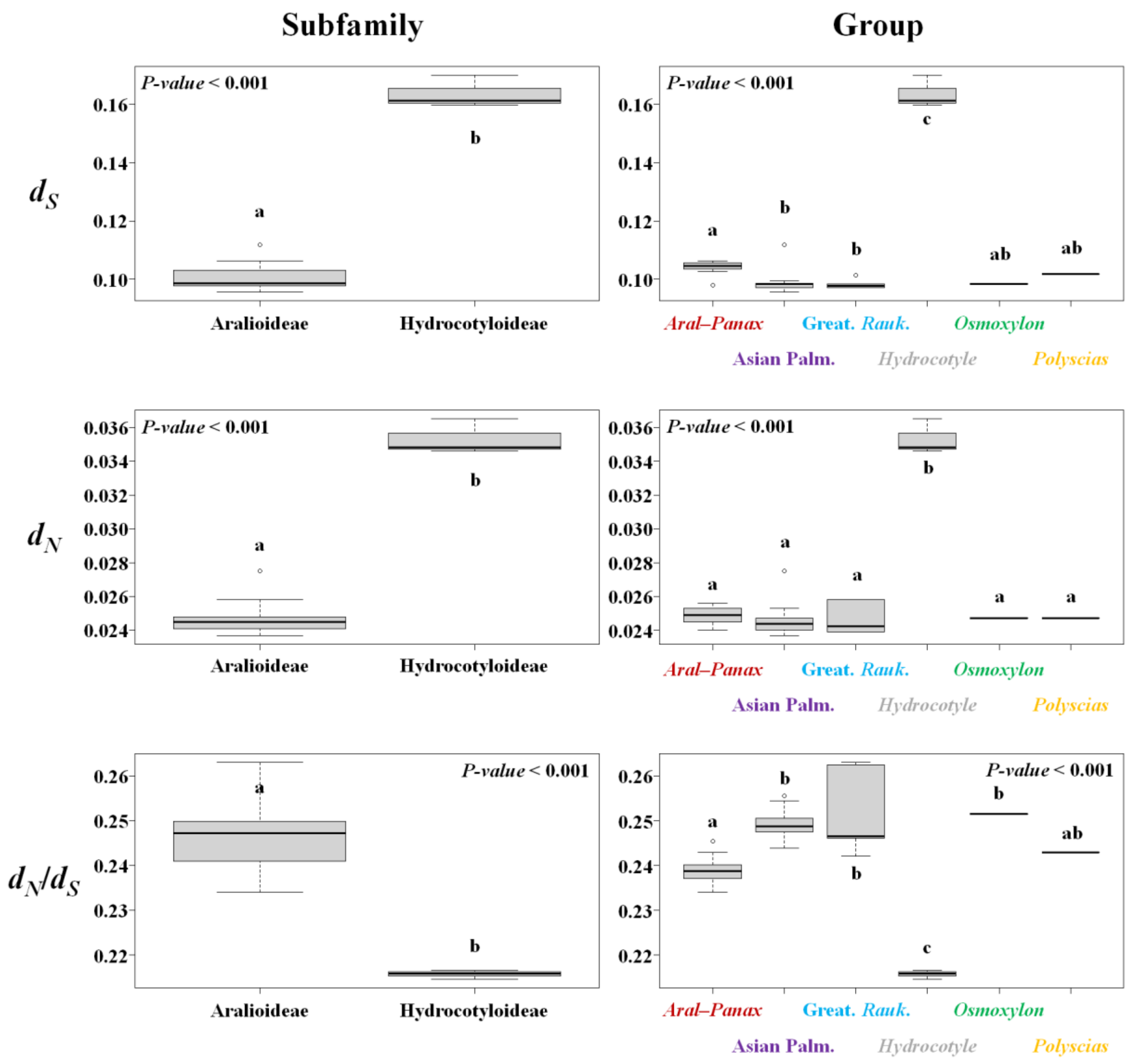

Supplementary Fig. S6. Box plots of nucleotide substitution rates from 78 plastid-coding genes of Araliaceae species. The plastid genome sequence of *Centella asiatica* (Apiaceae) was used as a reference to estimate synonymous (d_S) and nonsynonymous (d_N) substitution rates. Significant differences were analyzed by ANOVA and Bonferroni *post-hoc* tests in R. *Aral–Panax*: *Aralia–Panax* group; Asian Palm.: Asian Palmate group; Great. *Rauk*.: Greater *Raukaua* group.

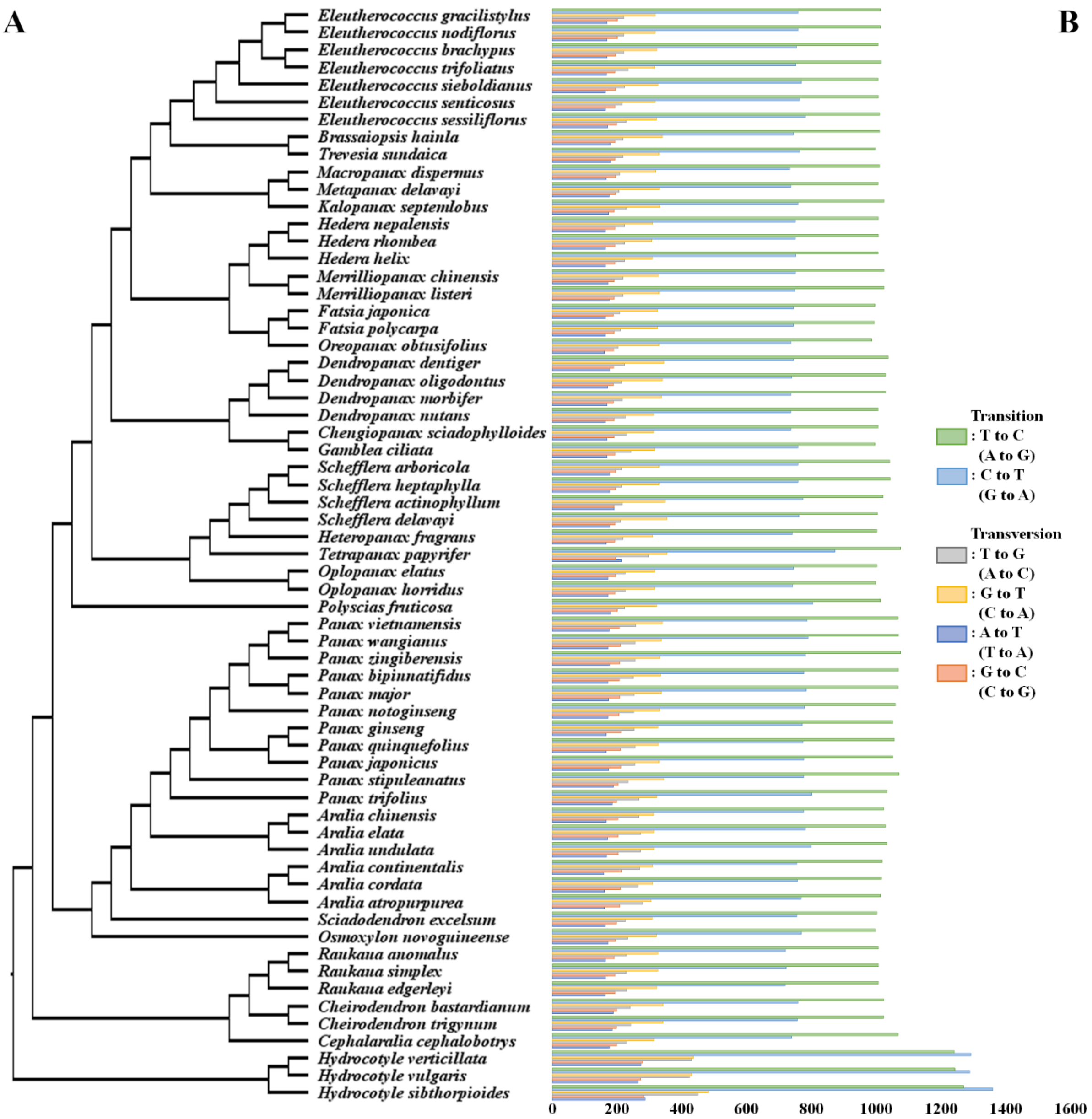

Supplementary Fig. S7. Accelerated AT-biased mutation in *Hydrocotyle* plastid genomes compared with other Araliaceae plastid genomes. (A) Cladogram of Araliaceae species adopted from the species network based on 66 plastome data (Figure 1). (B) Nucleotide substitution type analysis. A total of 78 protein-coding genes were used. Plastome data of *C. asiatica* were used as a standard.

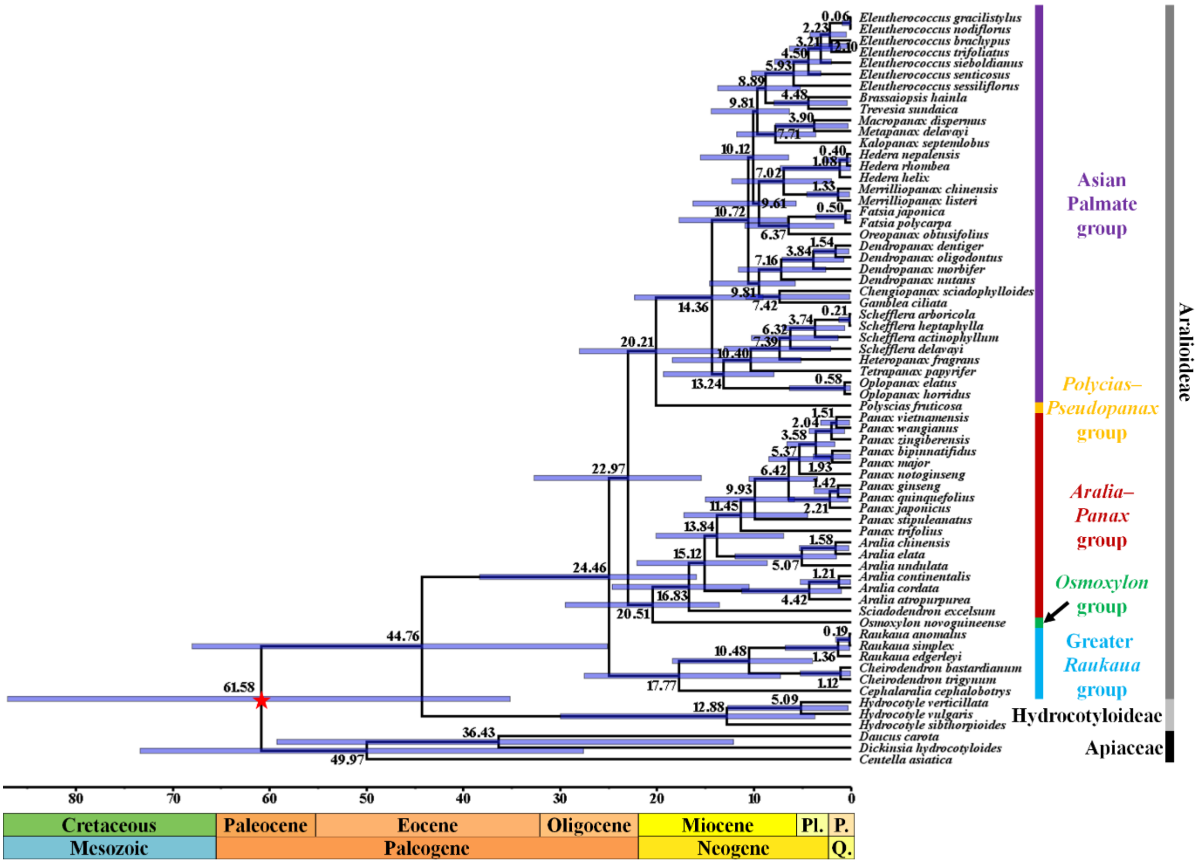

Supplementary Fig. S8. Chronogram of the Araliaceae inferred using BEAST. Divergence time estimation for Araliaceae species. A total of 78 protein-coding gene sequences were used. The red star indicates the calibration point.
